# Neuronal Stem Cells from Late-Onset Alzheimer Patients Show Altered Regulation of Sirtuin 1 Depending on Apolipoprotein E Indicating Disturbed Stem Cell Plasticity

**DOI:** 10.1007/s12035-023-03633-z

**Published:** 2023-09-20

**Authors:** Matthias Jung, Juliane-Susanne Jung, Jenny Pfeifer, Carla Hartmann, Toni Ehrhardt, Chaudhry Luqman Abid, Jenny Kintzel, Anne Puls, Anne Navarrete Santos, Thomas Hollemann, Dagmar Riemann, Dan Rujescu

**Affiliations:** 1 Institute of Physiological Chemistry (IPC), Faculty of Medicine, Martin Luther University Halle-Wittenberg, Hollystrasse 1, 06114 Halle (Saale), Germany; 2 Institute of Anatomy and Cell Biology, Faculty of Medicine, Martin Luther University Halle-Wittenberg, Grosse Steinstrasse 52, 06118 Halle (Saale), Germany; 3 Department Medical Immunology, Faculty of Medicine, Martin Luther University Halle-Wittenberg, Magdeburger Strasse 2, 06112 Halle (Saale), Germany; 4 Department of Psychiatry and Psychotherapy, Division of General Psychiatry, Medical University of Vienna, Währinger Gürtel 18-20, 1090 Vienna, Austria

**Keywords:** Late-onset Alzheimer’s diseases (AD), Apolipoprotein E isoform 4 (APOE4), Neural stem cell (NSC) plasticity, Sirtuin 1 (SIRT1), Telomere length

## Abstract

Late-onset Alzheimer’s disease (AD) is a complex multifactorial disease. The greatest known risk factor for late-onset AD is the E4 allele of the apolipoprotein E (APOE), while increasing age is the greatest known non-genetic risk factor. The cell type-specific functions of neural stem cells (NSCs), in particular their stem cell plasticity, remain poorly explored in the context of AD pathology. Here, we describe a new model that employs late-onset AD patient-derived induced pluripotent stem cells (iPSCs) to generate NSCs and to examine the role played by APOE4 in the expression of aging markers such as sirtuin 1 (SIRT1) in comparison to healthy subjects carrying APOE3. The effect of aging was investigated by using iPSC-derived NSCs from old age subjects as healthy matched controls. Transcript and protein analysis revealed that genes were expressed differently in NSCs from late-onset AD patients, e.g., exhibiting reduced autophagy-related protein 7 (ATG7), phosphatase and tensin homolog (PTEN), and fibroblast growth factor 2 (FGF2). Since SIRT1 expression differed between APOE3 and APOE4 NSCs, the suppression of APOE function in NSCs also repressed the expression of SIRT1. However, the forced expression of APOE3 by plasmids did not recover differently expressed genes. The altered aging markers indicate decreased plasticity of NSCs. Our study provides a suitable *in vitro* model to investigate changes in human NSCs associated with aging, APOE4, and late-onset AD.

**Supplementary Information:**

The online version contains supplementary material available at 10.1007/s12035-023-03633-z.

## Introduction

The significant increase in life expectancy over the last decades has led to aging societies worldwide. As a result, age-related diseases have become more prevalent, especially those affecting brain functions. The most common neuropsychiatric disorder is late-onset Alzheimer’s disease (AD), a complex multifactorial disease associated with genetic risk factors [1, 2]. To date, treatment of AD continues to be limited by the low therapeutic impact of available drugs intended to prevent or delay the disease onset. Many clinical trials have failed to understand AD pathology, prompting a search for alternative triggers and drivers of AD pathogenesis [3, 4]. Although the AD pathology is well characterized by neuronal accumulation of amyloid-β (Aβ) and tau fibrils [5], the specific cellular and molecular disease mechanisms responsible for disease onset are largely unknown. Accordingly, there is a growing interest to understand the preclinical stages of AD for the development of novel treatment approaches and to restore normal brain physiology.

The course of late-onset AD correlates with a decline in neurons and a loss of synaptic plasticity, which has been intensively studied using AD mouse models, through human post-mortem examinations, and by focusing on mild cognitive impairment (MCI) associated with AD [6–8]. The extracellular formation of Aβ plaques disrupts the communication between neurons while the intracellular formation of tau fibrils destabilizes the cytoskeleton and accelerates the loss of neurons [9, 10]. Accordingly, brain plasticity is reduced due to a lowered rejuvenation capacity and age-related resilience which combine with other AD risk factors including genetic susceptibility. Stem cell aging is linked with telomere damage and shortening [11]. In rodents, adult neuronal stem cells (NSCs) maintain their rejuvenation capacity and support resilience [12, 13], which could point toward the existence of similar mechanisms in the human brain [14]. In the adult human brain, NSCs are thought to reside in the subgranular zone in the dentate gyrus of the hippocampus and the ventricular-subventricular zone of the lateral ventricles [15]. However, neurogenesis is strongly restricted in the human brain and declines with both aging and the onset of neuropsychiatric diseases such as late-onset AD [16]. NSCs are generated during embryogenesis and represent neural precursors of radial glia cells that share many but not all properties with adult NSCs. Apolipoprotein E (APOE) is strongly expressed in developing and adult NSCs [17, 18]. In the adult brain, APOE plays an important role in lipid metabolism and is highly expressed in glia cells. DNA variations in the APOE gene generate different isoforms of APOE. The allele 4 (APOE4) dramatically changes the properties of the APOE protein. APOE4 represents the strongest genetic risk variant for late-onset AD [19] and related phenotypes, such as reduced hippocampal volume, suggest APOE4-dependent effects on NSCs [20]. However, the role of APOE4 in NSCs, especially within the context of aging and AD, remains poorly understood.

In this work, we used patient-specific induced pluripotent stem cells (iPSCs) to analyze aging markers, suggesting that NSC plasticity is altered in late-onset AD in an APOE4-dependent manner. Human iPSC-derived NSCs provide a powerful tool for the analysis of neuronal development, neuronal regeneration of the adult brain, and loss of regenerative capacities in aging and neurodegenerative diseases. First introduced by Yamanaka and colleagues [21], many labs now have the capacity to generate iPSCs. Recently, we and other researchers have developed *in vitro* models based on iPSCs carrying a certain donor-specific genetic background to better understand the related molecular and cellular pathways in neurodegenerative diseases [22–25]. However, there are currently no iPSC-based NSC models that carry APOE4. Here, we present an NSC model of APOE genetic variations which shows altered expressions of several aging markers such as autophagy-related 7 (ATG7), fibroblast growth factor 2 (FGF2), phosphatase and tensin homolog (PTEN), and sirtuin 1 (SIRT1), all of which depend on APOE4 in the context of late-onset AD. This model fills an existing gap in the field of neurophysiological modeling and allows to investigate the pathophysiological function of APOE variants in neurogenesis and aging.

## Materials and Methods

### Generation of iPSCs from B-LCLs

**Fig. 1:**
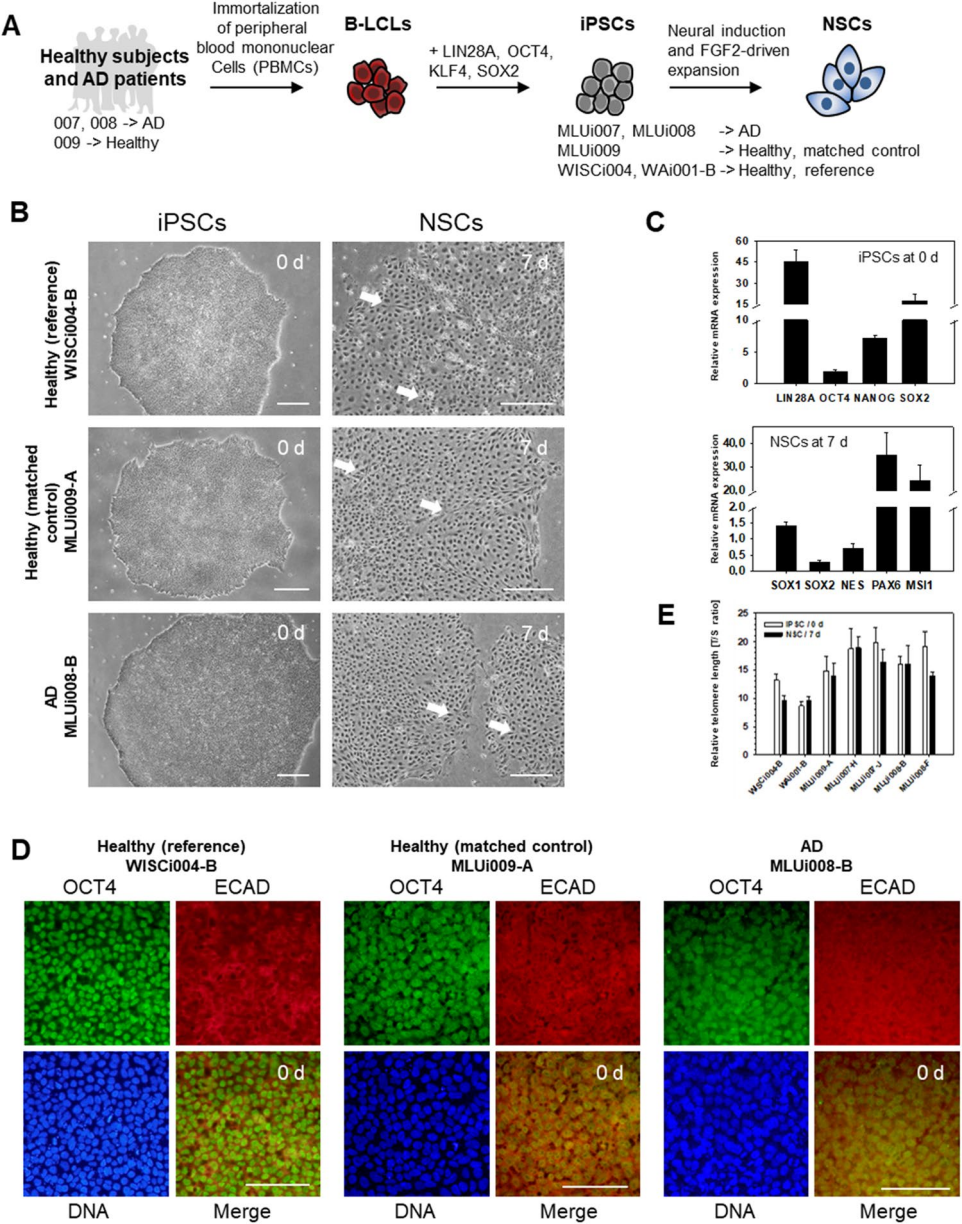
Characterization of iPSCs on 0 d and during neuronal differentiation on 7 d. **A** Scheme of modeling NSC differentiation: reference iPSC lines (WISCi004-B and WAi001-B), iPSC lines from one late-onset AD patient (MLUI007-J/H and MLUi008-B/F), and one iPSC line from one matched healthy control (MLUi009-A). **B** Phase contrast image for iPSCs and NSCs on 0 d and 7 d showing a homogenous differentiation into NSCs (scale bar left 300 μm; scale bar right 100 μm). White arrows indicate the rosette-like formation of NSCs representing a premature stage of neural rosettes. Data are shown for one iPSC line (clone) per donor. **C** Comparison of the three iPSC lines regarding their expression of pluripotency markers on 0 d and their expression of NSC markers on 7 d. We could prove the presence of pluripotency markers LIN28A, OCT4, NANOG, SOX2 in iPSCs and NSC markers SOX1, SOX2, NES, PAX6, MSI1 in NSCs. RT-PCR analysis by gel electrophoresis visualized as bar charts (one amplicon each from MLUi008-B, MLUi009-A, WISCi004-B was pooled for analysis; *n* = 3, mean ± SEM). **D** IF analysis on 0 d showed nuclear localization of OCT4 and cellular distribution of the epithelial marker ECAD in iPSCs (scale bar: 100 μm). **E** Telomere length measured by multiplex QRT-PCR in iPSCs on 0 d and in NSCs on 7 d; *N* = 3 differentiations, mean ± SEM

We collected whole blood samples and generated immortalized B-lymphoblastoid cell lines (B-LCLs) according to a recently published protocol [26]. B-LCLs were used for the generation of iPSC lines, namely, MLUi007-H, MLUi007-J, MLUi008-B, MLUi008-F, and MLUi009-A (Fig. 1A, using the ISSCR naming conventions [27]). B-LCLs were grown at 37.0°C in a humidified environment of 20.0% O_2_ and 5.0% CO_2_ (normoxia) and transferred to 5.0% O_2_, 5.0% CO_2_, and 90.0% N_2_ (hypoxia) for reprogramming and iPSC culture. Obtained iPSCs were cultured in mTeSR™1 (Stemcell Technologies) supplemented with 1.0% 10,000 U/10,000 μg penicillin/streptomycin (Thermo Fisher Scientific) on Matrigel™ (VWR) using 0.5 mg in 6.0 ml DMEM (Thermo Fisher Scientific) for coating. For iPSC generation, we modified a recently published protocol [28] adding treatment with 1 μM BIX-01294 and 0.04 μM RG-108 (both from Merck).

The MLUi009-A cell line was obtained from a healthy 64-year-old donor carrying homozygous APOE3. MLUi007-H and MLUi007-J were obtained from a 76-year-old late-onset AD patient carrying homozygous APOE4. MLUi008-B and MLUi008-F were obtained from a 79-year-old late-onset AD patient carrying APOE3 and APOE4 (Table 1). These cell lines are listed in the Human Pluripotent Stem Cell Registry (hPSCreg.eu). Both AD patients were recruited at the memory clinic of the Department of Psychiatry, LMU Munich, Germany, and late-onset AD was diagnosed according to the NINCDS-ADRDA criteria [29]. Genomic integrity of all iPSC lines was checked with the hPSC Genetic Analysis Kit (Stemcell Technologies) and through karyotyping including G-banding. Pluripotency was checked by the expression of pluripotency markers, and differentiation capacity was proofed by trilineage differentiation into derivatives of the three germ layers: ectoderm, endoderm, and mesoderm. The APOE genotype was identified in patient blood samples using the TaqMan™ SNP Genotyping Assay (Thermo Fisher Scientific) according to the manufacturer’s protocol. The APOE genotype was confirmed in the lines after reprogramming as per previous publications. A more detailed analysis of MLUi007-J and MLUi008-B has been published recently [30]. Reference iPSC lines, WISCi004-B and WAi001-B, were purchased from WiCell Research Institute.

**Table 1:** iPSCs to examine APOE4 and aging markers in the context of late-onset AD

hPSCreg name	Origin/donor	Experimental group Healthy/AD	Experimental group APOE3/APOE4	APOE status
MLUi009-A	Healthy subject 009/matched control	Healthy	APOE3	APOE33
WISCi004-B	Reference iPSC lines			APOE33
WAi001-B			APOE4	APOE43
MLUi008-B	AD patient 008	AD		APOE43
MLUi008-F				APOE43
MLUi007-H	AD patient 007			APOE44
MLUi007-J				APOE44

### Neuronal Differentiation of Donor-Specific iPSCs

All iPSCs were treated with collagenase IV (Thermo Fisher Scientific) for passaging and seeded on Matrigel™ in mTeSR™1 medium. For neuronal differentiation, all iPSCs cultured in Stemdiff™ neural induction medium (Stemcell Technologies) in a humidified environment of 20.0% O_2_ and 5.0% CO_2_ (normoxia). Neural induction medium was changed every other day. NSCs were harvested after day 7 (7 d).

### Transcript Analysis

RNA extraction was performed with RNeasy™ Mini Kit (Qiagen) according to the manufacturer’s protocol. 1.0 μg of total RNA was transcribed into cDNA by using Revertaid™ M-MuLV RT with buffer, RNase inhibitor, oligo (dT) 18 primers, and deoxyribonucleotide triphosphate (dNTPs; all from Thermo Fisher Scientific). Standard reverse transcriptase PCR (RT-PCR) analysis was performed with 1.0 μl template cDNA in a 25.0 μl reaction, 10x buffer BD, 25 mM MgCl_2_, 2.5 mM dNTPs, 5.0 U/μl Firepol™ DNA polymerase (all from Solis Biodyne) and 10 pmol/μl of each primer (Biomers). Quantitative real-time PCR (QRT-PCR) analyses were performed in triplicate with 1.0 μl template cDNA in a 25.0 μl reaction, Hot Firepol™ Evagreen™ QPCR Mix Plus, and 10 pmol/μl of each primer (Biomers). Relative QRT-PCR results were analyzed by the relative standard curve method. Each transcript amount was first calculated with a standard curve and then normalized to glyceraldehyde-3-phosphate dehydrogenase (GAPDH). Primers are listed in Table 2.

**Table 2:** List of primers for transcript analysis

Primer	Forward	Reverse	Accession no.
APOE	AGACACTGTCTGAGCAGGTG	GGGGTCAGTTGTTCCTCCAG	NM_001302688.2
ATG7	TCCTGGGCTCATCGCTTTTT	AGTCCTGGACGACTCACAGT	NM_006395.3
FGF2	GTGCTAACCGTTACCTGGCT	CAGTGCCACATACCAACTGG	NM_002006.6
GAPDH	ACGACCAAATCCGTTGACTC	ACAGTCAGCCGCATCTTCTT	NM_002046.7
LIN28	CCCCCCAGTGGATGTCTTT	CCGGAACCCTTCCATGTG	NM_024674.6
MSI 1	TGACCAAGAGATCCAGGGGT	CGATTGCGCCAGCACTTTAT	NM_002442.4
NANOG	AAATCTAAGAGGTGGCAGAAAAACA	CTTCTGCGTCACACCATTGC	NM_024865.4
NES	TTCCCTCAGCTTTCAGGACCC	GGACTGGGAGCAAAGATCCAA	NM_006617.2
OCT4	AGTTTGTGCCAGGGTTTTTG	TTGTGTTCCCAATTCCTTCC	NM_002701.6
p21	GCGACTGTGATGCGCTAATG	GAAGGTAGAGCTTGGGCAGG	NM_000389.5
PAX6	ACCCATTATCCAGATGTGTTTGCCCGAG	ATGGTGAAGCTGGGCATAGGCGGCAG	NM_000280.6
PRRX1	GACCATGACCTCCAGCTACG	GAGCAGGACGAGGTACGATG	NM_006902.5
PTEN	AGTTCCCTCAGCCGTTACCT	AGGTTTCCTCTGGTCCTGGT	NM_000314.8
SIRT1	TCCAAAACTGTGGCAGCTAA	CGAGTGCTCTCCCCACATA	NM_012238.5
SOX1	AGCAGTGTCGCTCCAATTCA	ACGATGAGTGTTACCTTGGC	NM_005986.3
SOX2	CACTGCCCCTCTCACACATG	CCCATTTCCCTCGTTTTTCTT	NM_003106.4
SOX17	TAGTTGGGGTGGTCCTGCAT	CGCTTTCATGGTGTGGGCTA	NM_022454.4
STAT3	CGGCGTCCAGTTCACTACTA	ATCCTCTGAGAGCTGCAACG	NM_001384993.1

### Telomere Length Analysis

Monochrome multiplex QRT-PCR for measuring the telomere/single copy gene (T/S) ratio was performed and analyzed as previously described [31]. In brief, genomic DNA was extracted from NSCs using DNeasy™ Blood and Tissue Kit (Qiagen) according to the manufacturer’s instructions. Monochrome multiplex QRT-PCR was performed with 20.0 ng genomic DNA, 900 nM of each telomere primer, 500 nM of each albumin primer (reference gene), and Hot Firepol™ Evagreen™ QPCR Mix Plus. Primers are listed in Table 3.

**Table 3:** List of primers for telomere length analysis

Primer	Forward	Reverse	Reference
Telomere	CGGCGGCGGGCGGCGCGGGCTGGGCGGAAATGCTGCACAGAATCCTTG	GCCCGGCCCGCCGCGCCCGTCCCGCCGGAAAAGCATGGTCGCCTGTT	[31]
ALB	GCCCGGCCCGCCGCGCCCGTCCCGCCGGAAAAGCATGGTCGCCTGTT	CGGCCCGCCGCGCCCGTCCCGCCGGAGGAGAAGTCTGCCGTT	[31]

### Immunofluorescence (IF) Analysis

Human iPSCs and NSCs were fixed on coverslips with 4.0% paraformaldehyde in phosphate-buffered saline (PBS). Samples were permeabilized for 30 min with 1.0% horse serum and 0.02% Trtion™X-100. Then, cells were treated with 3.5% horse serum for 30 min. Coverslips were washed with PBS and incubated with primary antibodies (Table 4) overnight at 4.0°C in the dark. Coverslips were washed again with PBS and incubated with a fluorescent dye-labeled secondary antibody (Table 4) for 2 h at room temperature in the dark. For DNA staining, coverslips were washed with PBS, incubated with Hoechst 33342™ (Thermo Fisher Scientific) for 5 min at room temperature, and then washed in deionized water. Immunofluorescence staining was visualized using a Keyence BZ-8100E microscope.

**Table 4:** List of antibodies for WB, IF, and flow cytometry analysis

Name	Application, dilution	Supplier	Catalog number
Mouse IgG anti-human ACTB	WB: 1:40,000	Merck	A5441
Mouse IgG anti-human APOE	WB: 1:500; IF: 1:500	Bio-Techne	NB110-60531
Mouse IgG anti-human ATG7	WB: 1:1000; IF: 1:100	Bio-Techne	MAB6608
Mouse IgG anti-human COL1A1	IF: 1:100	Merck	C2456
Rabbit IgG anti-human CDH1	IF: 1:100	Abcam	Ab40772
Mouse IgG anti-human FGF2	WB: 1:100	Santa Cruz	sc-136255
Mouse IgG anti-human FGF2	IF: 1:100	Bio-Techne	NBP1-47749
Rabbit IgG anti-human MSI1	IF: 1:100	Merck	AB5977
Mouse IgG anti-human NES	IF: 1:100	Santa Cruz	Sc-23927
Rabbit IgG anti-human NEUROG3	IF: 1:100	Abcam	Ab38548
Mouse IgG anti-human OCT4 (POU5F1)	IF: 1:100	Santa Cruz	Sc-5279
Mouse IgG anti-human PAX6	IF: 1:100	Santa Cruz	Sc-53108
Goat IgG anti-human PRRX1	IF: 1:100	Bio-Techne	NBP1-06067
Rabbit IgG anti-human p21m (CDKN1A)	WB: 1:1000; IF: 1:800	Cell Signaling Technology	2947
Mouse IgG anti-human p16 (CDKN2A)	IF: 1:100	Origene	TA500036
Mouse IgM anti-human PTEN	WB: 1:100; IF: 1:200	Thermo Fisher Scientific	MA5-12278
Mouse IgG anti-human SIRT1	WB: 1:1000; IF: 1:1000	Bio-Techne	NBP1-51641
Goat IgG anti-human SOX2	IF: 1:100	Santa Cruz	Sc17320
Mouse IgG anti-human SOX17	IF: 1:100	Santa Cruz	Sc-130295
Mouse IgM anti-human SSEA1	IF: 1:100	Santa Cruz	Sc-21702
Mouse IgG anti-human STAT3	WB: 1:5000; IF: 1:100	Thermo Fisher Scientific	MA1-13042
Mouse IgG anti-human VIM	IF: 1:100	Merck	MAB3400
Goat IgG anti-mouse HRP	WB: 1:10,000	Dianova	115-035-003
Goat IgG anti-rabbit HRP	WB: 1:3000	Cell Signaling Technology	7074
Goat IgM anti-mouse HRP	WB: 1:4000	Thermo Fisher Scientific	62-6820
Donkey IgG anti-goat Alexa Fluor™ 488	IF: 1:400	Thermo Fisher Scientific	A11055
Goat IgG anti-mouse Alexa Fluor™ 488	IF: 1:400	Thermo Fisher Scientific	A11001
Goat IgG anti-rabbit Alexa Fluor™ 488	IF: 1:400	Thermo Fisher Scientific	A11034
Rabbit IgG anti-rat Alexa Fluor™ 488	IF: 1:400	Thermo Fisher Scientific	A21210
Goat IgG anti-mouse Cy™3	IF: 1:400	Dianova	115-165-003
Goat IgG anti-mouse IgM Cy™3	IF: 1:400	Dianova	115-165-075
Mouse IgG anti-human NES-Alexa Fluor™ 488	FC: 1:50	Stemcell Technologies	60091AD.1
Recombinant human IgG anti-PAX6-PE	FC: 1:11	Miltenyi Biotec	130-107-828
Recombinant human IgG anti-SOX1-FITC	FC: 1:50	Miltenyi Biotec	130-111-157
Recombinant human IgG anti-SOX2-FITC	FC: 1:11	Miltenyi Biotec	130-104-993
Mouse IgG isotype control Alexa Fluor™ 488	FC: 1:50	Stemcell Technologies	60070AD.1
Recombinant human IgG isotype control PE	FC: 1:11	Miltenyi Biotec	130-104-613
Recombinant human IgG isotype control FITC	FC: 1:50	Miltenyi Biotec	130-104-611

*WB* western blot analysis, *IF* immunofluorescence analysis, *FC* flow cytometry, *HRP* horseradish peroxidase

### Western Blot (WB) Analysis

Cells were lysed in cell extraction buffer, incubated on ice for 30 min, and centrifuged with 13,000 rpm at 4°C. Cell extraction buffer consisted of 100 mM Tris, 100 mM Triton™ X-100, 1.0% NaCl, 1 mM EDTA, 10.0% glycerol, 1 mM EGTA, 0.1% SDS, 0.5% sodium deoxycholate (all from Carl Roth), phosphatase inhibitor, and protease inhibitor (both Roche). Sodium dodecyl sulfate polyacrylamide gel electrophoresis was used to separate 30 μg of protein and immunoblot them on nitrocellulose membranes (Cytiva). Unspecific binding was blocked by 3.0% bovine serum albumin in tris-buffered saline plus Tween™20 (TBST) (all from Carl Roth). Membranes were incubated overnight with primary antibodies at 4°C, washed in TBST, incubated with horseradish peroxidase (HRP)-labeled secondary antibodies for 90 min at room temperature, and washed again (Table 4). Protein was visualized with enhanced chemiluminescence substrate for HRP using a ChemiDoc XRS imager (Bio-Rad). Protein amounts were quantified as ratio band intensities with Image Lab 6.0 software (Bio-Rad) and normalized with beta-actin (ACTB) in the same blot.

### Flow Cytometry

Quantification of neuronal markers was performed using a BD FACS Canto II flow cytometer (BD Biosciences). 2×10^5^ NSCs were fixed and permeabilized using the FoxP3 staining buffer set (Miltenyi Biotec) according to manufacturer’s instructions. The cell suspension was incubated with fluorescence-labeled primary antibody on ice for 30 min (Table 4). The data were processed using the Cell Quest Pro™ software (BD Biosciences) which yielded histograms showing the number of positive cells in comparison to isotype controls.

### Induction of APOE3 and Repression of APOE

On 7 d, NSCs were detached with Accutase™ (Merck) and seeded with a density of 300,000 cells/cm^2^ onto Matrigel™-coated dishes in Stemdiff™ neural progenitor medium (Stemcell Technologies). For APOE3 induction, NSCs with a confluence of approximately 60.0% were transfected with pCMV4-APOE3 (Addgene) using Viromer™ red (Lipocalyx) according to the manufacturer’s protocol. For APOE repression, NSCs were transfected with a pool of APOE siRNAs (Merck) using Viromer™ blue (Lipocalyx) according to manufacturer’s protocol. Mock transfections served as controls.

### Statistical Analysis

Data were analyzed using Sigma Plot 12.3 (Systat Software) to identify statistically significant differences between the analyzed groups (*p* < 0.05). If the QRT-PCR and WB data were normally distributed, a one-way ANOVA was used, otherwise a Kruskal-Wallis one-way ANOVA on ranks. The data from plasmid and siRNA experiments were normally distributed; Student’s *t*-test was used. Data are presented as mean ± standard error of the mean (mean ± SEM).

## Results

### Generation and Characterization of NSCs from Late-Onset AD Patients

**Fig. 2:**
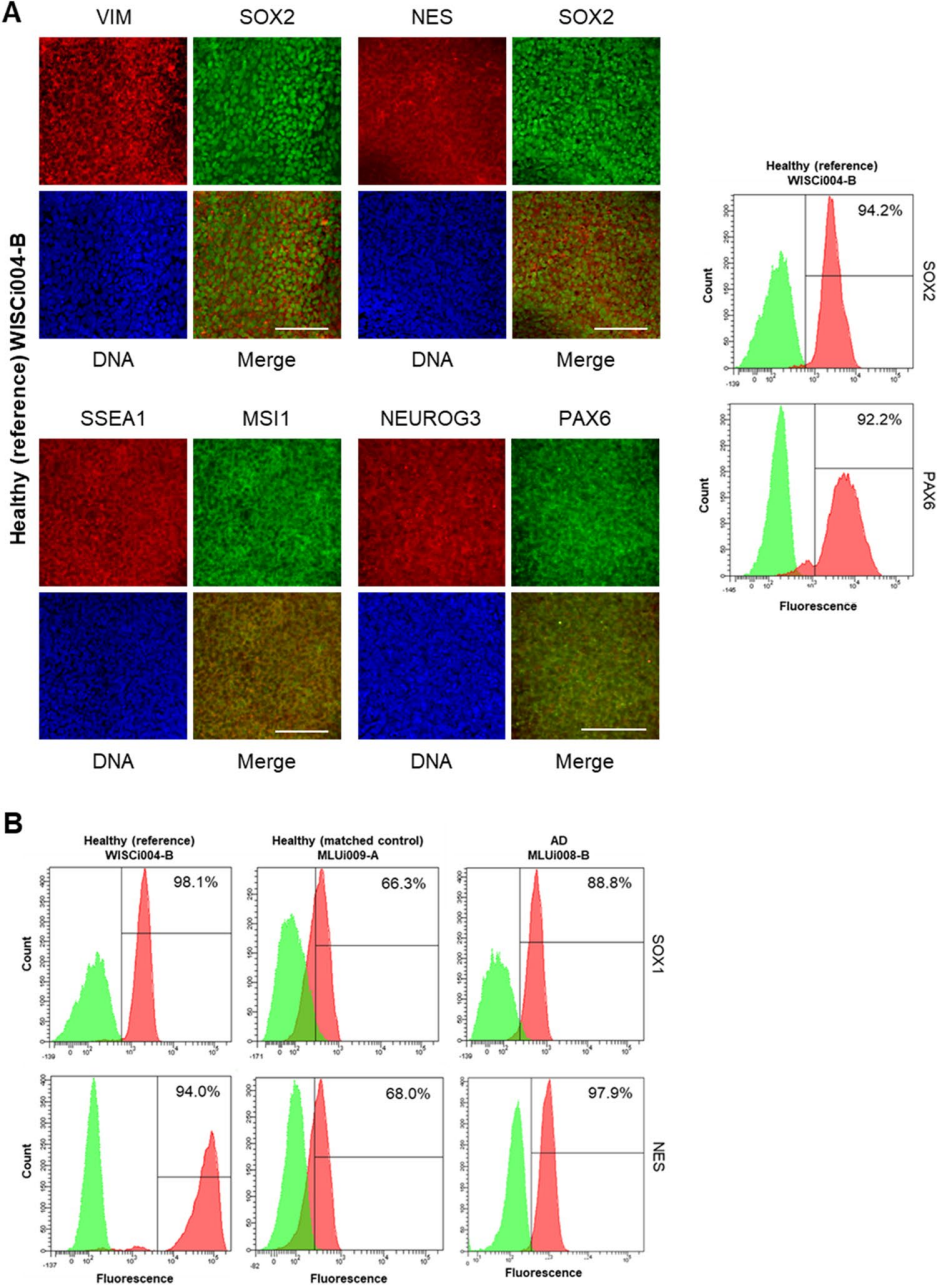
Profiling of neuronal markers in iPSC-derived NSCs on 7 d. **A** NSCs from healthy (reference) WISCi004-B analyzed by immunofluorescence (IF) and flow cytometry. IF analysis showed the localization of neural stem cell (NSC) markers SRY-box transcription factor 2 (SOX2), vimentin (VIM), nestin (NES), paired box 6 (PAX6), neurogenin 3 (NEUROG3), Musashi RNA-binding protein 1 (MSI1), and the epitope stage-specific embryonic antigen (SSEA1). Proteins of interest are shown by co-staining in green and red versus DNA staining in blue (scale bar, 100 μm). Flow cytometry histograms are shown for neuronal markers SOX2 and PAX6 (each in red; isotype controls in green). **B** Flow cytometry histograms of neuronal markers in NSCs from WICi004-B (reference), MLUi009-A (matched control), and MLUi008-B (AD) on 7 d proving the induction of NSC markers SOX1 and NES (each in red; isotype controls in green). Representative histograms show positive counts versus fluorescence intensity. The percentage of positive cells is indicated

To characterize NSC plasticity in the context of late-onset AD, we set up an *in vitro* model with iPSCs undergoing early neuronal differentiation (Fig. 1A), using AD iPSCs (MLUi007-H/J and MLUi008-B/F) and healthy controls (MLUi009-A, WISCi004-B [32], and WAi001-B [33]) (Table 1). These iPSCs were subjected to neuronal induction in monolayer cultures and characterized on 0 d and 7 d. MLUi007-J, MLUi008-B, and MLUi009-A were checked for pluripotency, differentiation capacity (Fig. S1), and genomic integrity (Fig. S1–2). Undifferentiated iPSCs showed the typical cobblestone morphology on 0 d (Fig. 1B left) and the transcription of the pluripotency markers Lin-28 homolog A (LIN28A), Nanog homeobox (NANOG), POU class 5 homeobox 1 (POUF5F1 alias OCT4), and SRY-box transcription factor 2 (SOX2) could be confirmed (Fig. 1C, top). Data are shown for one iPSC line of the three different origins AD, healthy (matched control), and healthy (reference). Further material for other iPS cell lines is provided in the supplements (Fig. S3). On 7 d of neuronal differentiation, NSC populations had formed typical rosette-like patterns (Fig. 1B right, white arrows). Furthermore, we observed the induction of early neuronal markers Musashi RNA-binding protein 1 (MSI1), nestin (NES), paired box 6 (PAX6), SRY-box transcription factor 2 (SOX1), and SOX2 on 7 d (Fig. 1C, bottom). Through IF analysis, we were able to confirm protein expression of pluripotency on 0 d and neuronal markers on 7 d. OCT4 protein localization was verified in both AD iPSCs (MLUi008-B) and healthy controls (WISCi004-B and MLUi009-A). We found that OCT4 localizes in the nucleus and the epithelial marker cadherin 1 (CDH1 alias ECAD) localizes in the cytoplasm (Fig. 1D). We verified protein expression and cellular localization of NSC markers MSI1, NES, neurogenin 3 (NEUROG3), PAX6, SOX2, stage-specific embryonic antigen (SSEA1), and vimentin (VIM) in WISCi004-B on 7 d (Fig. 1A). SOX2 protein was detected in the nucleus, whereas other marker proteins localized predominantly within the cytoplasm of NSCs (Fig. 2A). Furthermore, flow cytometry analysis revealed that in WISCi004-B almost all cells expressed SOX2 and PAX6 on 7 d (Fig. 2A). The comparably strong induction of SOX1 and NES in NSCs from different cell lines (Fig. 2B) is a strong indication of the similarity of the generated NSCs, which allowed us to perform further comparative analyses regarding aging markers. We analyzed telomere lengths to monitor the plasticity of iPSCs and found differences on 0 d in the above cell lines. Due to differentiation, we detected significant telomere shortening in some NSCs between 0 and 7 days (Fig. 1E).

### Aging Markers in NSCs from Late-Onset AD Patients

**Fig. 3:**
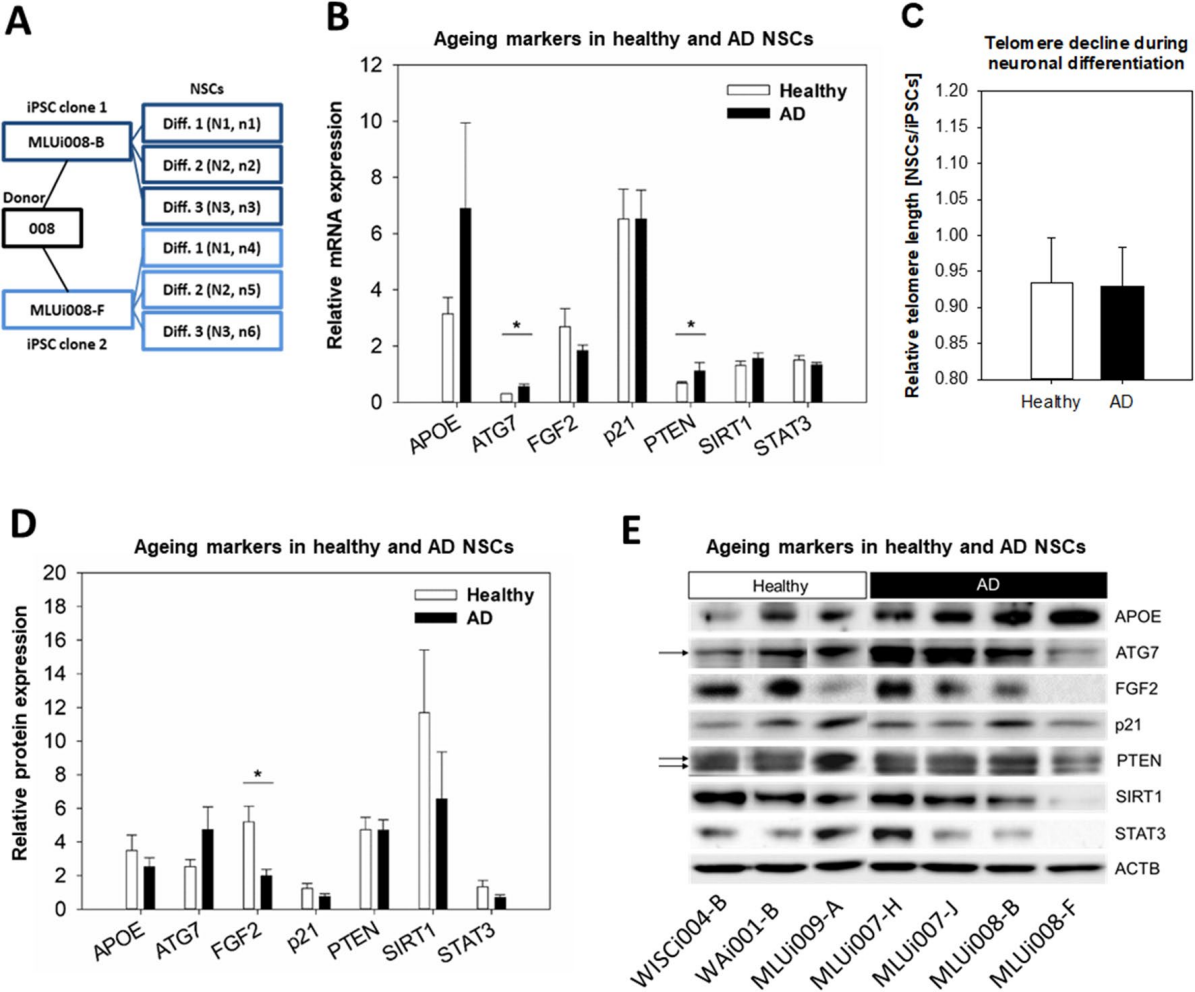
Aging markers in NSCs are affected by late-onset AD. **A** Scheme illustrating the sample collection of NSCs from two iPSC clones of the same donor. Quantification of **B** transcript and **D** protein amounts of the aging markers APOE, ATG7, FGF2, p21, PTEN, SIRT1, and STAT3. Results are shown as mean ± SEM (*p = 0.05). The bar charts compare three healthy iPSCs (WISCi004-B, WAi001-B, and MLUi009-A) versus four AD iPSCs (MLUi007-H/J and MLUi008-B/F) on 7 d, which were differentiated three times into NSCs (*N* = 3 differentiations; healthy: *n* = 9, AD: *n* = 12). **E** Representative image of WB membranes. **C** Telomere length measured by multiplex QRT-PCR in iPSCs on 0 d and in NSCs on 7 d, shown as the ratio NSCs/iPSCs grouped according to health status (*N* = 3 differentiations; healthy: *n* = 9, AD: *n* = 12). Results are shown as mean ± SEM (*p = 0.05)

**Fig. 4:**
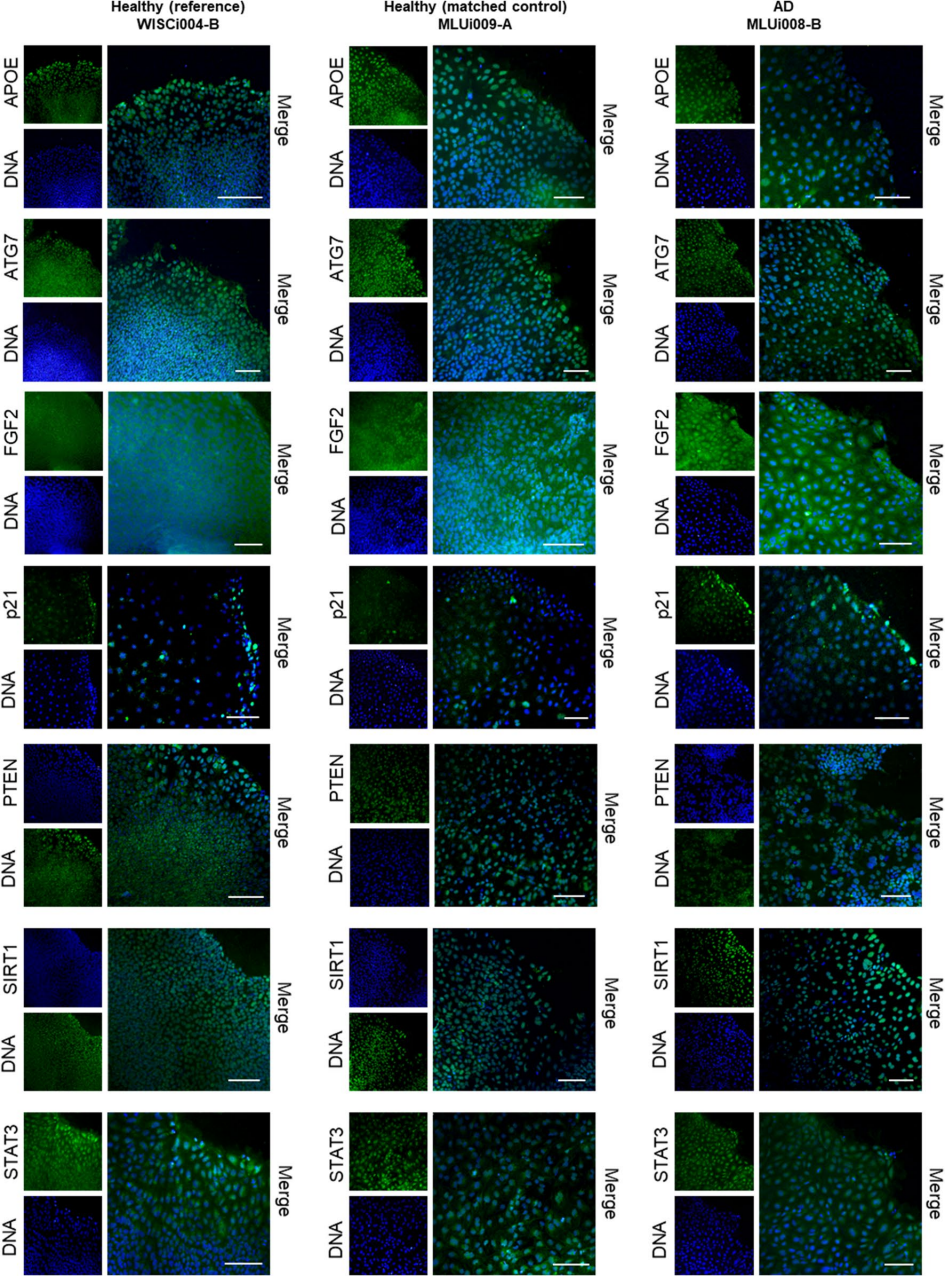
Detection of marker proteins for cellular aging in NSCs through immunofluorescence (IF). IF analysis showed cellular localization of APOE, ATG7, FGF2, p21, PTEN, SIRT1, and STAT3. Protein of interest is shown in green versus DNA staining in blue (scale bar, 100 μm)

As aging contributes to reduced NSC plasticity, we analyzed aging markers in NSCs derived from iPSCs generated from both late-onset AD patients and healthy controls (Table 1). Samples consisted of NSCs from two iPSC clones of the same donor (Fig. 3A). The aging markers APOE, ATG7, FGF2, p21, PTEN, SIRT1, and STAT3 were analyzed on the mRNA and protein level. Transcripts of ATG7 and PTEN were higher in the AD group (Fig. 3B), although these differences were not significant at the protein level (Fig. 3D, E). Healthy controls had a more than twofold higher level of the proliferative marker FGF2 than the AD group (Fig. 3D, E, Fig. S4). FGF2 expression varies between different cell lines and between different replicate differentiations, even when NSC markers are induced at high levels. Both the subcellular localization of the transcription factors and the cytoplasmic distribution of structural proteins were confirmed by IF analysis (Fig. 4). Collagen type I alpha 1 chain (COL1A1) and cyclin-dependent kinase inhibitor 2A (CDKN2A alias p16), both regulating stem cell proliferation, were detected abundantly in NSCs by IF analysis (Fig. S5).

To compare the decrease in telomere length between healthy controls and AD during neuronal differentiation of iPSCs into NSCs from 0 d to 7 d (Table 1), we plotted the telomere length (calculated as the T/S ratio; TS) of NSCs relative to iPSCs, with shortening corresponding to TS_NSCs_/TS_iPSCs_ < 1. We found no significant differences between healthy controls and AD (Fig. 3C).

### SIRT1 Is an APOE4-Dependent Aging Marker in NSCs

**Fig. 5:**
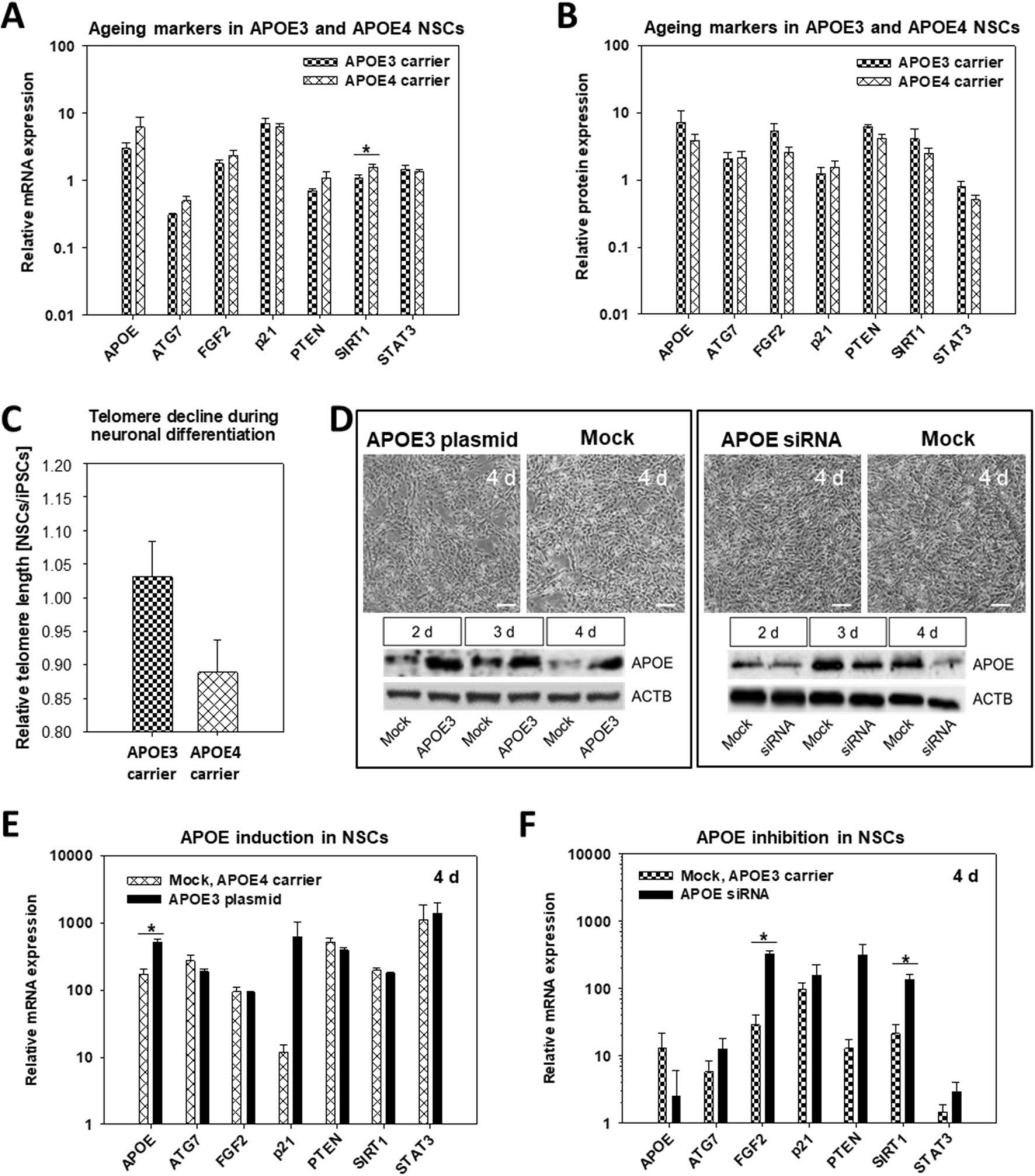
Aging markers in NSCs are affected by APOE genotype. **A** Transcript and **B** protein expression analysis of the aging markers APOE, ATG7, FGF2, p21, PTEN, SIRT1, and STAT3. Results are shown as mean ± SEM (*p = 0.05). The bar charts compare two iPSCs carrying APOE3 (WISCi004-B and MLUi009-A) versus five iPSCs carrying APOE4 (WAi001-B, MLUi007-H/J and MLUi008-B/F) on 7 d, which were differentiated three times into NSCs (*N* = 3 differentiations; APOE3: *n* = 6, APOE4: *n* = 15). **C** Telomere length measured by multiplex QRT-PCR in iPSCs on 0 d and in NSCs on 7 d, shown as the ratio NSCs/iPSCs grouped according to APOE status (*N* = 3 differentiations; APOE3: *n* = 6, APOE4: *n* = 15). Results are shown as mean ± SEM (*p = 0.05). **D** Manipulation of APOE gene expression by APOE siRNAs and APOE3 plasmids in NSCs generated from reference cell line WISCi004-B on 7 d. No morphological changes are shown by phase contrast imaging up to 4 d after transfection. WB analysis showed the strongest APOE protein repression by APOE siRNAs and strongest APOE induction by APOE3 plasmids on 4 d. Representative images of stained WB membranes are shown (scale bar, 100 μm). Transcript analysis of aging markers in NSCs on 4 d after **E** APOE inhibition and **F** APOE induction. Results are shown as mean ± SEM (*p = 0.05). For APOE inhibition, MLUi009-A (healthy matched control, APOE3 carrier) was differentiated into NSCs and transfected with siRNAs on 7 d (*N* = 3 differentiations; mock: *n* = 3, siRNA: *n* = 3). For APOE induction, the MLUi007-H from (AD, APOE4 carrier) was differentiated into NSCs and transfected with APOE3 plasmids at 7 d (*N* = 3 differentiations; mock: *n* = 3, APOE3 plasmids: *n* = 3)

To further investigate the impact of the APOE4 isoform on aging marker expression, we compared the NSC samples of APOE genotypes APOE3 and APOE4 (Table 1). APOE3 carriers showed expression levels comparable to healthy donors while expression levels in APOE4 carriers were more similar to diseased donors. The only significant (*p* = 0.047) change was a 27.3% increase in SIRT1 transcripts (Fig. 5A) while SIRT1 protein expression was not significantly (*p* = 0.080) altered (Fig. 5B). Further material for the WB analysis is provided in the supplements (Fig. S6). Comparing the decrease in telomere length between APOE3 and APOE4 carriers during neuronal differentiation of iPSCs into NSCs from 0 d to 7 d, we found no significant differences (Fig. 5C).

To assess the impact of APOE4 on NSCs, we performed loss- and gain-of-function experiments of APOE and analyzed SIRT1 along with aging markers. Recently, APOE4-related phenotypes could be generated through gene editing of APOE3 [34], suggesting that reduced functional amounts of APOE protein or reduced functionality of the APOE4 isoform can be ameliorated by the transfection of plasmids for the forced expression of APOE3. Accordingly, we transfected APOE3 plasmids into NSCs derived from WISCi004-B. While this did not have any adverse effects on NSC morphology, the APOE protein was strongly enriched in NSCs by 4 d (Fig. 5D; Fig. S7). Furthermore, transfection of the APOE3 plasmid caused a slight and temporary induction of SIRT1 protein expression that had resolved by 4 d (Fig. S8). This result is in good agreement with our previous observation of a non-significant tendency to stabilize SIRT1 protein in APOE3 carriers (Fig. 5B).

Further, we employed NSCs from AD MLUi007-H (APOE4 carrier) for transcriptional analysis of a set of aging markers in NSCs on 4 d after APOE3 plasmid transfection. However, we did not find any significant change in aging marker expression including SIRT1 (Fig. 5E). Thus, APOE3 plasmid transfection could not recover the elevated SIRT1 transcription observed in APOE4 carriers (Fig. 5A, E).

Previous studies suggested that the APOE4 isoform was subject to structural alterations [35, 36] which explained its reduced functionality. The isoform-specific loss-of-function in APOE can be mimicked by the transfection of APOE siRNAs. Accordingly, we established transfection APOE siRNAs into WISCi004-B-derived NSCs and observed no adverse effects on NSC morphology after 4 d (Fig. 5D) but found that APOE protein was consistently reduced in NSCs after 4 d (Fig. 5D; Fig. S7), as was SIRT1 protein (Fig. S8). This observation in is good agreement with our earlier finding of a non-significant tendency for reduced SIRT1 protein expression in APOE4 carriers (Fig. 5B). Lastly, we analyzed the transcript levels of all aging markers in NSCs on 4 d from healthy MLUi009-A (APOE3 carriers) upon APOE siRNA transfection, which revealed significantly elevated FGF2 and SIRT1 transcripts (Fig. 5F). As in APOE4 carriers, APOE siRNA strongly increased SIRT1 transcription in APOE3 genetic background (Fig. 5A, F).

## Discussion

### Altered Aging Markers in NSCs in the Context of Late-Onset AD

In various stem cells types, autophagy is a fundamental survival mechanism responsible for stem cell quiescence, activation, differentiation, and self-renewal [37]. While animal models have shown a decline of ATG7 with increasing age [38], we found elevated ATG7 transcript levels in AD NSCs. Within the context of AD and other neurodegenerative diseases, ATG7 is required during autophagy for autophagosome assembly. Aβ degradation enzymes and autophagy are the main Aβ clearance pathways [39]. The observed upregulation of ATG7 could be due to an upregulation of the autophagy program as suggested by the amyloid cascade hypothesis, which postulates an accumulation of extracellular Aβ as one of the causes of late-onset AD [40]. Moreover, recent findings in murine NSCs demonstrated that autophagy is involved in autophagy-mediated cell death induced by apoptosis in response to chronic restraint stress [41]. These significant differences in ATG7 mRNA expression between healthy and AD could not be confirmed when grouped by APOE genotype, although the same tendency remained (Fig. 3B; Fig. 5A). Neither APOE induction nor inhibition significantly affected ATG7 transcript levels (Fig. 5E, F).

PTEN is a cell cycle-regulating molecule and controls cell growth inhibition. We found an increased PTEN transcription in AD NSCs. This is in agreement with previous studies on both cellular and animal models, which demonstrated that Aβ causes increased PTEN levels with several effects on neurogenesis [42] and linked increased neuronal PTEN to synaptic depression in neurons [43]. Further, PTEN expression was used as a predictor for the conversion of mild cognitive impairment (MCI) to AD [44]. The present study suggests that elevated PTEN may be a potential prodromal factor in AD that could regulate NSC plasticity.

ATG7 and PTEN were significantly increased on the mRNA level, but not significant altered on the protein level suggesting that translation was regulated by microRNAs (miRs) as recently described in NSCs [45, 46].

We showed expression of COL1A1 in NSCs, an extracellular matrix protein that is involved in proliferation of various stem cells. Mutations in COL1A1 have been associated with hydrocephaly suggesting an important role in the development of the neocortex rather than NSC development [47].

While earlier studies described the AD-dependent relevance of cyclin-dependent kinase inhibitor 1A (CDKN1A alias p21) and STAT3 [48, 49], we found no significant differences between AD NSCs and healthy NSCs. This supports previous findings in mice which showed that p21 may serve as an important negative regulator of the proliferation in neural progenitors but not in NSCs [50]. We found that CDKN2A is expressed in iPSC-derived NSCs, but recent studies showed increased CDKN2A in AD iPSC-derived neurons suggesting a minor role in AD NSCs similar to CDKN1A [51]. Signal transducer and activator of transcription 3 (STAT3) is linked with neuroinflammation and Aβ production over the course of AD, but a recent study suggests that STAT3 dysregulation may play a larger role in microglia that are not derived from NSCs [52].

Our data showed reduced FGF2 protein in AD NSCs (Fig. 3D). Similarly reduced FGF2 levels and affected FGF2 pathways have been reported for *post-mortem* examinations of human brains from late-onset AD patients [53]. Aβ is known to affect neurogenesis in AD. Furthermore, damaged neurons secrete FGF2 to enhance microglial migration and phagocytosis [54]. Accordingly, many studies have focused on FGF2 treatment as a way to enhance synaptic functions. For instance, in mice, FGF2 treatment improved the functionality of both neurons and glia [55]. FGF2 is a well-known driver of NSC maintenance and development during embryogenesis and in adults. The number of FGF2-expressing astrocytes decreases with increasing age and this may be due to reduced FGF2 in the hippocampus [56]. While synaptic plasticity is regulated by FGF2 via neurite growth, axonal branching, and enhancement of long-term potentiation, the underlying mechanisms are still not fully understood [57].

### Altered APOE in NSCs and the Role of APOE4

Our data showed no significant differences in mRNA and protein levels of APOE between healthy and AD NSCs (Fig. 3B, D) nor between NSCs carrying APOE3 and APOE4 (Fig. 5A, B). Earlier studies examining post-mortem brains of late-onset AD patients APOE levels found both normal and reduced APOE levels in certain brain regions including the hippocampus [58, 59]. We hypothesize that APOE levels depend on age, the genetic APOE status, and the brain region, which is important especially for NSC plasticity in the hippocampus.

In AD, dysregulated APOE has been linked to general protein aggregation, mitochondrial dysfunction, and disturbed lipid and glucose metabolisms which affect neurons throughout their entire life span from formation, over maturation, through aging [60]. We found APOE mRNA and protein present in all analyzed NSCs in agreement with recent findings [61]. A recent study investigated an APOE knockout in murine NSCs and demonstrated that functional APOE is crucial for neurogenesis [62]. Likely, the impact of APOE on NSCs is also mediated by direct transcriptional effects. In addition, the APOE protein undergoes nuclear localization and binds to the SIRT1 promoter [63]. There is evidence that APOE interacts with the RE1 silencing transcription factor, a transcriptional repressor crucial for proper neurogenesis [64]. Accordingly, we could proof the presence of APOE in the nucleus of NSCs.

APOE4 is a supportive characteristic to distinguish phenotypically healthy elderly from those who need treatment to prevent the onset of AD. MCI is an important prodromal factor for AD [65] whose predictive power is improved by stratifying subjects according to their APOE4 status. MCI is likely the result of decreased neuronal plasticity. During aging, stem cell plasticity appears to be impaired [66]. An imbalance between firing homeostasis and synaptic plasticity could be a driving force into the early phase of AD [7]. Therefore, adult NSCs could be a target for preventive therapies, as they are crucial for the maintenance of neuronal plasticity. However, little is known about the impact of altered APOE and the role of APOE4 in the context of aging markers in NSCs. APOE4 function and its effect on aging markers is better understood in mature cell types. For example, post-mortem studies in humans found that ATG7 was significantly altered in astrocytes from APOE4 carriers compared to APOE3 carriers [67].

Although many studies have been focused on APOE in human iPSC *in vitro* models [34], the vast majority focused on mature cells and neglected that APOE could have a large impact on NSCs.

### APOE Status Impacts the Regulatory Enzyme SIRT1

SIRT1, also called silent information regulator 1, predominantly deacetylates histones to open DNA, thereby inhibiting DNA transcription. Sirtuin family proteins (SIRTs) are defined as class III histone deacetylases. We could prove the nuclear localization of SIRT1 in NSCs at 7 d (Fig. 4). We compared SIRT1 in NSCs of APOE3 and APOE4 carriers and found that the APOE4 genotype was associated with elevated levels of SIRT1 transcripts SIRT1 (Table 5). SIRT1 was even more induced by the transfection of NSCs with siRNA against APOE. WB analysis revealed that SIRT1 protein decreased when APOE expression was suppressed, and SIRT1 protein was stably expressed when APOE3 was overexpressed. This observation agrees with findings for patients with Parkinson’s disease whose NSCs showed a stress-induced reduction in SIRT1 protein [68]. Interestingly, recent publications demonstrated an impact of SIRT1 on aging via NAD metabolism and age-related diseases like progeria [69]. SIRT1 regulates pathways responsible for NSC maintenance and differentiation including cell cycle progression [70], autophagy in neurons [71], and neurodegeneration as described for NSCs in Parkinson’s disease [68]. Interestingly, SIRT1 is a target of metformin, a medication for type 2 diabetes that has also been hypothesized to have preventive effects on AD progression. In old mice, metformin treatment was described to act in an APOE genotype-dependent manner [72]. Accordingly, SIRT1 has become a therapeutic focal point in drug development for age-related diseases including AD [73, 74].

**Table 5:** Significant mRNA expression changes observed in aging markers

	Healthy/AD	APOE3/AOPE4	APOE siRNA	APOE3 plasmid
APOE	-	-	-	x
ATG7	x	-	-	-
FGF2	x	-	x	-
p21	-	-	-	-
PTEN	x	-	-	-
SIRT1	-	x	x	-
STAT3	-	-	-	-

-: no change; x: significant change

Apart from histones, SIRT1 can also deacetylate other proteins such as p53. Due to its enormous influence on transcriptional processes, SIRT1 can regulate cellular processes involved in molecular aging including DNA repair, autophagy, and inflammation in the brain [75]. In senescence, nuclear SIRT1 is an autophagy substrate. This pathway contributes to the loss of SIRT1 as tissues age. Mechanistically, SIRT1 can drive the production of metabolites linked to APP processing, aging, and neurogenesis in the brain [76].

Although there is significant evidence for a link between APOE and SIRT1, it remains unclear whether and how these two molecules interact. For example, a small molecule appears sufficient to increase memory and SIRT1 protein levels in the hippocampus of E4FAD mice with an APOE4-related genetic background [77]. At a cellular level, SIRT1 regulates macronutrient selection in the central nervous system and peripheral tissues [78]. In the context of healthy aging, SIRT1 influences the health-promoting effects of caloric restriction in neurons by shifting diet choice from sucrose to fat. We propose that APOE, as key regulator of fat metabolism, acts as a potential mediator of nutrient and metabolic sensing including SIRT1 in the neuronal development that governs networks in the central nervous system and extending to its periphery.

**Fig. 6:**
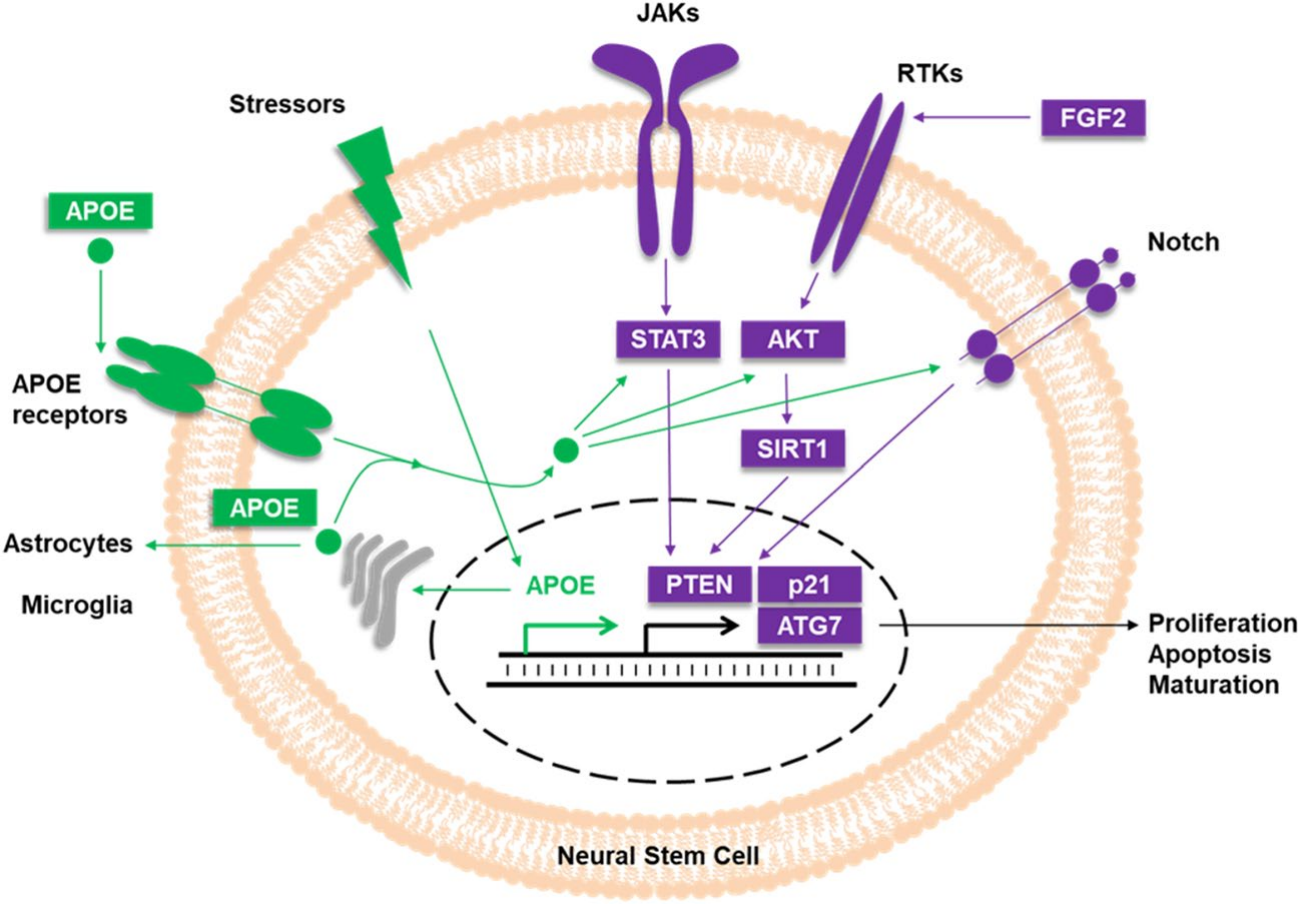
Proposed mechanisms for APOE and its impact on aging markers for regulating NSC plasticity. Observed effects on PTEN, ATG7, FGF2, and SIRT1 may reflect the impact of APOE on RTK signaling including FGF2 as key signaling molecule, SIRT1 as transcriptional regulator, and PTEN and ATG7 as target genes. RTKs: receptor tyrosine kinases; JAKs: Janus kinases; AKT: protein kinases B; gray: Golgi apparatus; green arrows: APOE signaling; violet arrows: aging markers signaling via JAKs, RTKs, and Notch

The suppression of APOE by siRNA led to a significant increase of SIRT1 and FGF2 mRNA (Fig. 5F). Both SIRT1 and FGF2 have been shown to regulate the cell fate of NSCs [79, 80]. At the protein level, however, FGF2 was significantly reduced in AD and not significantly altered in APOE4 carriers. SIRT1 protein was also consistently reduced by APOE siRNA. One possible explanation for these seemingly contradictory findings is the major impact of APOE4 on signaling pathways that include SIRT1 downstream of receptor tyrosine kinases (RTK) of the FGF2 signaling pathway (Fig. 6). SIRT1 and FGF2 protein amount might also be influenced by their individual protein stability or translational efficiency [81, 82]. Further, translation of SIRT1 and FGF2 is regulated by certain miRs. Recently, miR-153 increased adult neurogenesis in the hippocampus and improved the cognitive abilities of aged mice [83]. SIRT1 is a predicted target of mmu-miR-153-5p. In a different study, SIRT1 was already confirmed as a target of miR-135a whereby the lower SIRT1 levels were associated with decreased reprogramming efficiency from mouse embryonic fibroblasts to iPSCs [84]. While several studies have shown a link between SIRT1, FGF2, and APOE in stem cells, additional mechanistic studies are needed to elucidate how they interact to regulate NSC plasticity. In particular, the role of SIRT1 in DNA repair, DNA methylation, and histone modification may provide further insights into the crosstalk of SIRT1 and APOE.

We aimed to rescue the APOE4 phenotype by overexpression of APOE3. However, the forced expression of APOE3 in NSCs carrying APOE4 had no significant effect on FGF2 and SIRT1 transcript levels (Fig. 5E). Most likely, NSCs still produce APOE4 which competes with APOE3 or even acts as a dominant-negative APOE form. This could be driven by APOE aggregation, since unlipidated APOE protein monomers have been shown to form multimers such as dimers, and APOE can even aggregate to form fibrils [85]. Furthermore, APOE4 is much less lipidated than APOE3, leading to increased aggregate formation. Such a correlation was also shown in AD patients [86].

Other studies have found more contrasting results, e.g., that APOE4 (rather than APOE3) reduced neuroprotective SIRT1 on mRNA and protein levels [74]. These different findings with regard to the effects of APOE4 on SIRT1 may result from the use of different cellular models and experimental designs. Our data suggest that induced SIRT1 mRNA needs to be completed by other factors that are disturbed by APOE4 to accomplish the translation of neuroprotective SIRT1 protein in APOE4 NSCs.

### Telomere Length in NSCs Within the Context of Age and APOE4

Telomere shortening has been shown to co-occur with a strong reduction in SIRT1 and other sirtuins in mice [87], while FGF2 is a proven regulator of telomeres in human embryonic stem cells [88]. In our model, both SIRT1 and FGF2 were found to be regulated by APOE inhibition suggesting that APOE may indirectly regulate telomere length in NSCs. Shorter telomeres have been reported to be associated with age, AD, and APOE4 genotype [89].

### Altered Aging Markers in NSCs Within the Context of Aging and Rejuvenation

While we found altered aging markers in NSCs, these aging markers are not exclusively expressed in NSCs. Recently, we described the similar set of aging markers in mesenchymal stem cells [90] and suggested that shared signaling pathways may be present in different types of adult stem cells. Interestingly, aged mesenchymal stem cells also showed significant alterations for ATG7, FGF2, PTEN, and SIRT1 which suggests that aging markers have an impact on adult stem cells that goes beyond their context-specific interactions.

### Final Remarks

We verified NSC differentiation by the presence of developmental markers of NSCs in accordance with recent publications [91, 92]. Despite the large variety in published protocols including floating or adherent steps, neural induction follows the same developmental principles. It is important to highlight that our *in vitro* model is indeed a human model, because recent studies have shown that human iPSC models may provide insights that the corresponding animal studies failed to produce [93]. Nevertheless, care must be taken when interpreting the results from human *in vitro* NSC models as the NSC’s origin has a great effect on the responses, e.g., embryonic NSCs vs adult NSCs of the adult human brain, or NSCs derived from different neurogenic niches such as the subventricular zone of the lateral ventricles vs the subgranular zone of the hippocampal dentate gyrus [57].

### Supplementary Information

ESM 1 (DOCX 5.71 MB)

## Data Availability

The data supporting the findings of this study have been included in this article and the associated supplementary materials. The raw data can be obtained from the corresponding author upon reasonable request.

## Abbreviations

ACTB: Actin beta
AD: Alzheimer’s disease
Aβ: Amyloid-β
APOE: Apolipoprotein E
APOE4: Apolipoprotein E isoform 4
ATG7: Autophagy-related 7
B-LCLs: B-lymphoblastoid cell lines
CDH1 alias ECAD: Cadherin 1
COL1A1: Collagen type I alpha 1 chain
CDKN1A alias p21: Cyclin-dependent kinase inhibitor 1A
CDKN2A alias p16: Cyclin-dependent kinase inhibitor 2A
dNTP: Deoxyribonucleotide triphosphate
FGF2: Fibroblast growth factor 2
FC: Flow cytometry
GAPDH: Glyceraldehyde-3-phosphate dehydrogenase
HRP: Horseradish peroxidase
IF: Immunofluorescence
iPSCs: Induced pluripotent stem cells
LIN28A: Lin-28 homolog A
MCI: Mild cognitive impairment
NANOG: Nanog homeobox
NES: Nestin
NSCs: Neuronal stem cells
PAX 6: Paired box 6
PBMCs: Peripheral blood mononuclear cells
PTEN: Phosphatase and tensin homolog
PBS: Phosphate-buffered saline
POU5F1 alias OCT4: POU class 5 homeobox 1
STAT3: Signal transducer and activator of transcription 3
SIRT1: Sirtuin 1
SOX1, SOX2: SRY-box transcription factor 1 and 2
SEM: Standard error of the mean
TBST: Tris-buffered saline plus Tween™20
WB: Western blot
QRT-PCR: Quantitative real-time PCR
