# Supplementary material for: Neuronal Stem Cells from Late-Onset Alzheimer Patients Show Altered Regulation of Sirtuin 1 Depending on Apolipoprotein E Indicating Disturbed Stem Cell Plasticity
Source: Mol Neurobiol. 2023 Sep 20;61(3):1562–79. doi: 10.1007/s12035-023-03633-z (PMC10896791; doi:10.1007/s12035-023-03633-z)
Supplement: Supplementary file 1 — (DOCX 5.71 MB) [file 12035_2023_3633_MOESM1_ESM.docx]

**Journal: Molecular Neurobiology**

**Supplementary Information (SI)**

**Neuronal Stem Cells from Late-Onset Alzheimer Patients show Altered Regulation of Sirtuin 1 Depending on Apolipoprotein E Indicating Disturbed Stem Cell Plasticity.**

Matthias Jung^#1^, Juliane-Susanne Jung^#2^, Jenny Pfeifer^1^, Carla Hartmann^1^, Toni Ehrhardt^1^, Chaudhry Luqman Abid^1^, Jenny Kintzel^1^, Anne Puls^1^, Anne Navarrete Santos^2^, Thomas Hollemann^1^, Dagmar Riemann^3^, Dan Rujescu^4^

*^#^shared first authorship*

^1^Martin Luther University Halle-Wittenberg, Institute for Physiological Chemistry (IPC), Hollystrasse 1, 06114 Halle (Saale), Germany

^2^Martin Luther University Halle-Wittenberg, Department Anatomy and Cell Biology, Grosse Steinstrasse 52, 06118 Halle (Saale), Germany

^3^Martin Luther University Halle-Wittenberg, Department Medical Immunology, Magdeburger Strasse 2, 06118 Halle (Saale), Germany

^4^Department of Psychiatry and Psychotherapy, Division of General Psychiatry, Medical University of Vienna, Austria

**Corresponding author:**

Dr. Matthias Jung

Martin Luther University Halle-Wittenberg, Institute for Physiological Chemistry (IPC), Hollystrasse 1, 06114 Halle (Saale), Germany

email: [matthias.jung@uk-halle.de](mailto:matthias.jung@uk-halle.de)

phone: +49/345/3839


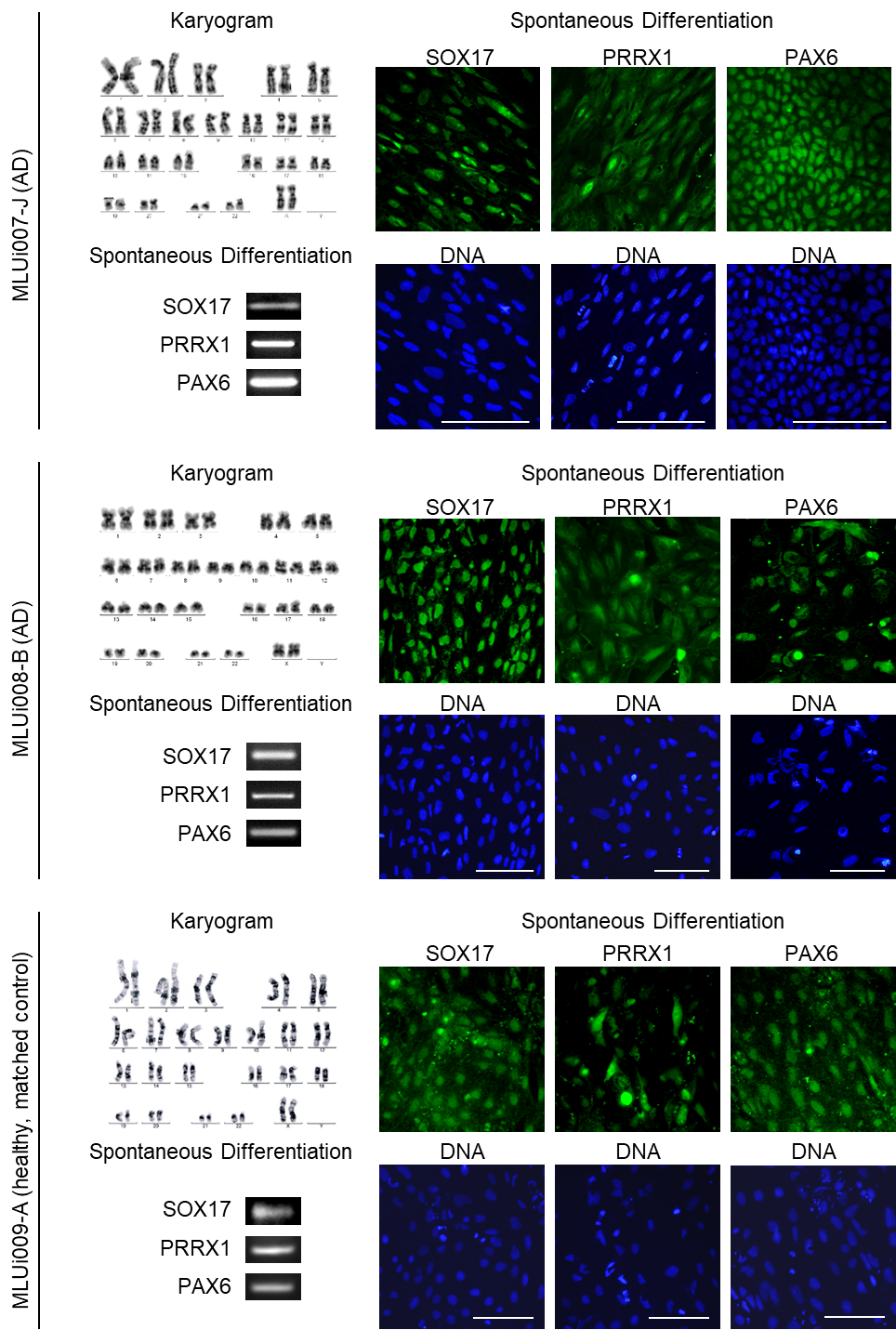


**Fig. S1** Characterization of genomic integrity and differentiation capacity of MLUi007‑J, MLUi008‑B, and MLUi009‑A**.** The analysis of 10-20 metaphases revealed no adverse effects on the karyotype indicating preserved genome integrity. Representative karyograms show a visible karyotype with condensed chromosomes and Giemsa banding. Spontaneous differentiation of induced pluripotent stem cells (iPSCs) demonstrated the induction into three germ layers mesoderm, endoderm, and ectoderm. Therefore, iPSCs were differentiated in DMEM/F12 containing 20% fetal calf serum and analyzed by transcript and IF analysis at 10 d for the presence of SRY‑box transcription factor 17 (SOX17; endoderm), Paired related homeobox 1 (PRRX1; mesoderm), and Paired box 6 (PAX6; ectoderm).


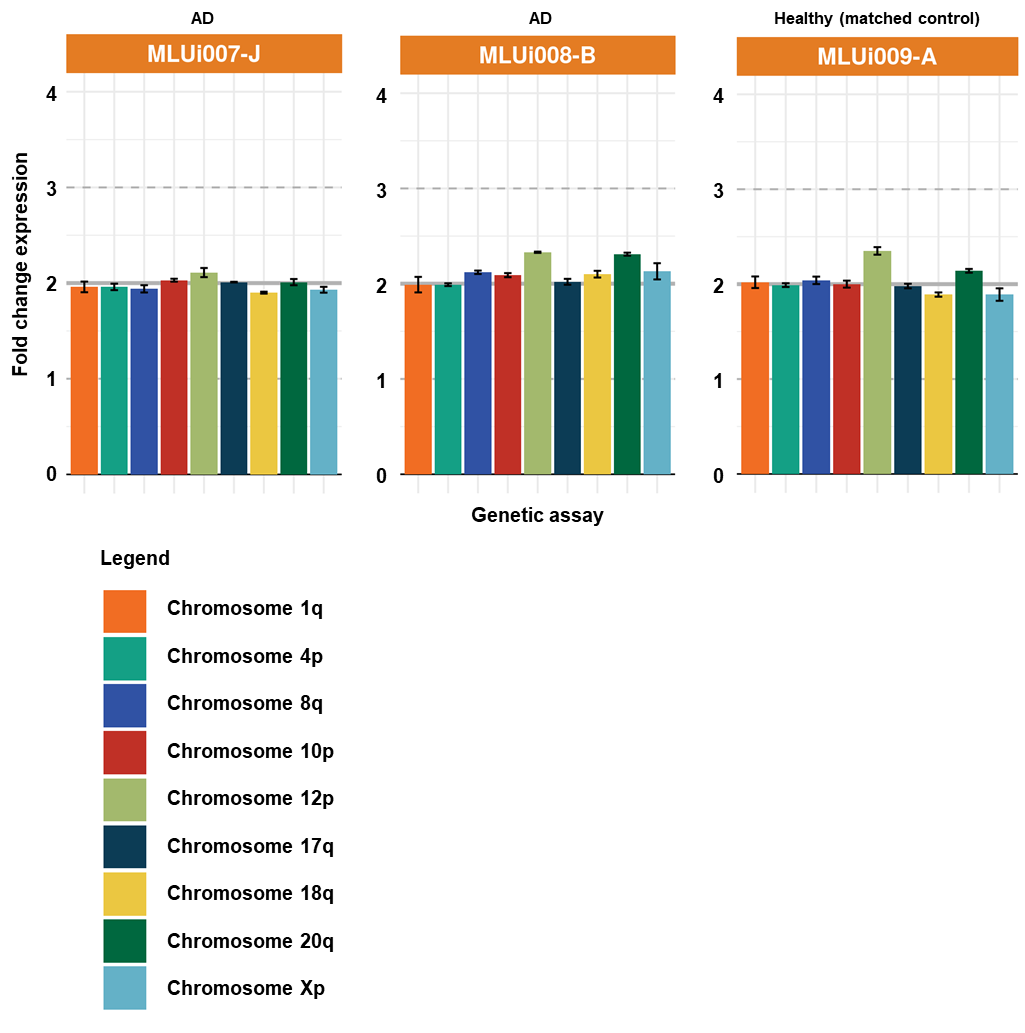


**Fig. S2** Monitoring of MLUi007‑J, MLUi008‑B, and MLUi009‑A for the eight most common karyotype abnormalities reported in pluripotent stem cells**. (A)** The genetic analysis is shown for induced pluripotent stem cells (iPSCs) obtained from patients (Alzheimer’s disease; AD) and healthy (matched donors). There were no adverse duplication or deletion in the stated chromosomal regions (see legend). These chromosomal regions were analyzed to detect the eight most common karyotype abnormalities reported in iPSCs.

**
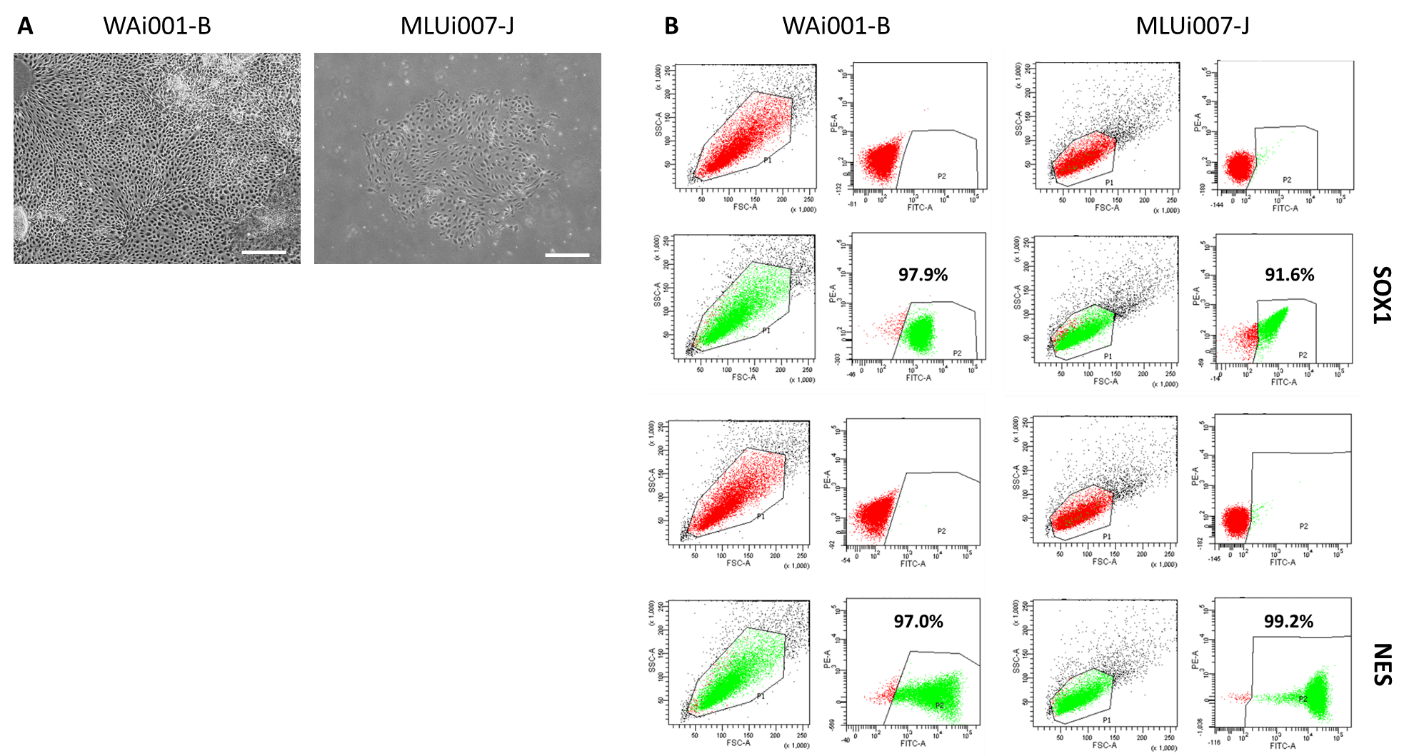
**

**
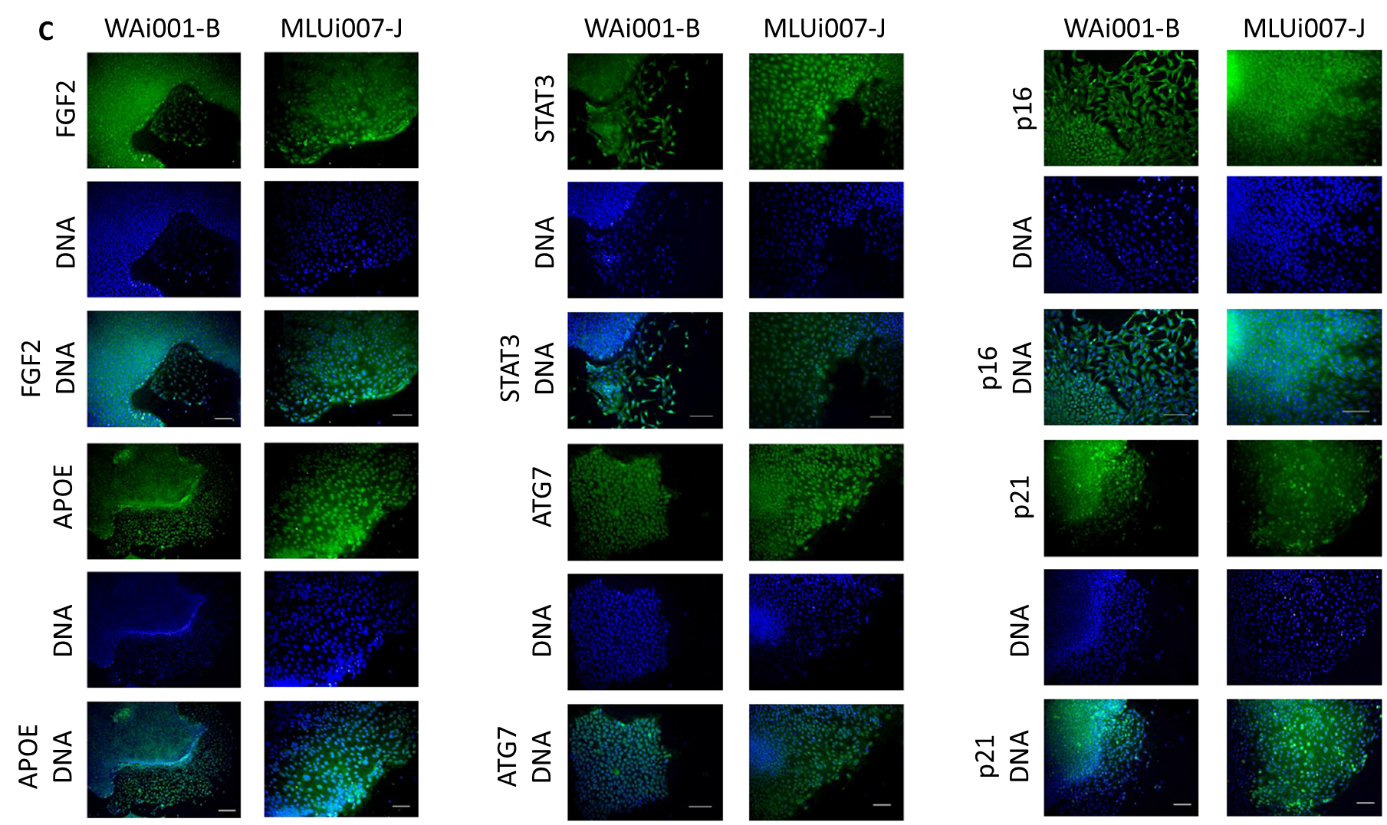
**

**
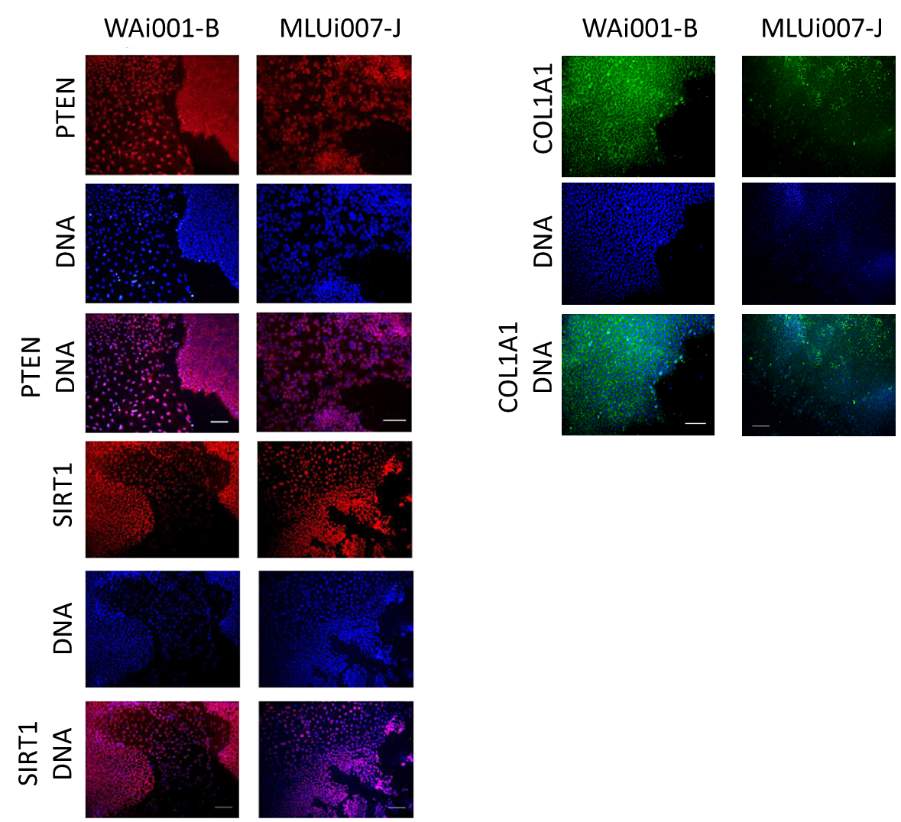
**

**Fig. S3** Supplemental data on the characterization of neural stem cells (NSCs) derived from WAi001-B and MLUi007-J. **(A)** Phase contrast images for NSCs on 7 d (scale bar: 100 µm). **(B)** Flow cytometry analysis of neuronal markers in NSCs on 7 d proving the induction of NSC markers SRY-box transcription factor 1 (SOX1) and nestin (NES; each in green; negative cells are red). Negative isotype controls are shown above staining. **(C)** Detection of marker proteins for cellular aging in NSCs through immunofluorescence (IF) analysis. IF analysis showed cellular localization of Apolipoprotein E (APOE), autophagy related protein 7 (ATG7), fibroblast growth factor 2 (FGF2), cyclin dependent kinase inhibitor 1A (CDKN1A alias p21), phosphatase and tensin homolog (PTEN), sirtuin 1 (SIRT1), and signal transducer and activator of transcription 3 (STAT3). Protein of interest is shown in green versus DNA staining in blue (scale bar: 100 µm).


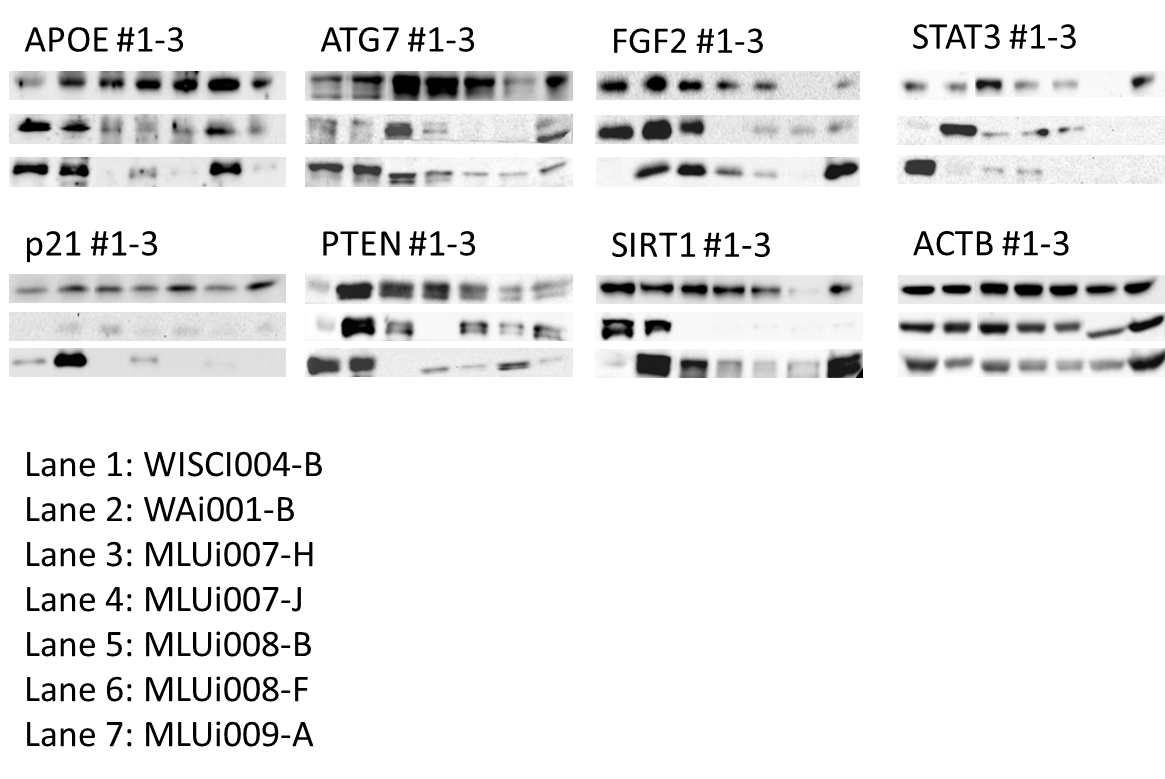


**Fig. S4** Images of WB membranes analysed in Fig. 3D and Fig. 5B.

**
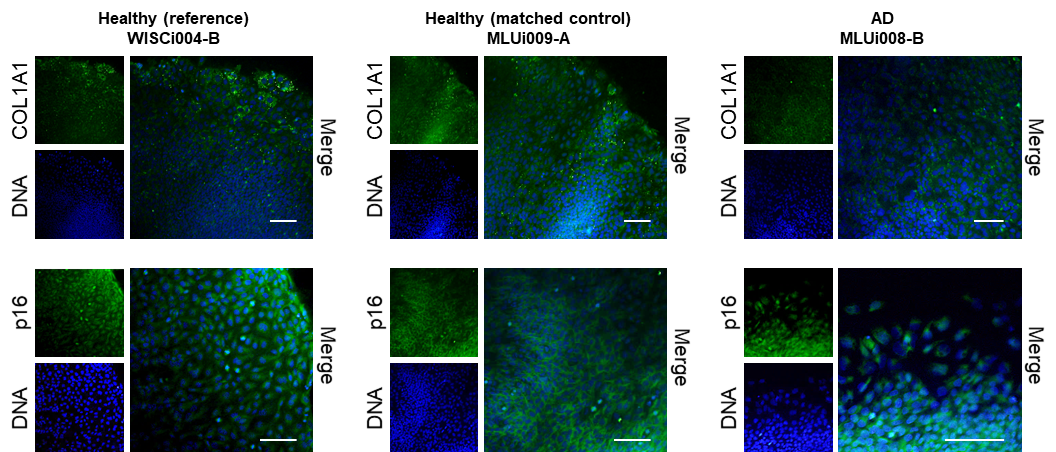
**

**Fig. S5** Cellular localization of additional ageing marker proteins in neural stem cells (NSCs). IF analysis verified protein expression of ageing markers collagen type I alpha 1 chain (COL1A1) and Cyclin dependent kinase inhibitor 2A (CDKN2A alias p16). Protein of interest is shown in green versus DNA staining in blue (scale bar: 100 µm). COL1A1 was present in the cytoplasm and between NSCs as part of the extracellular matrix. The p16 protein was present in the nucleus of some cells and broadly detectable in the cytoplasm across NSCs

**
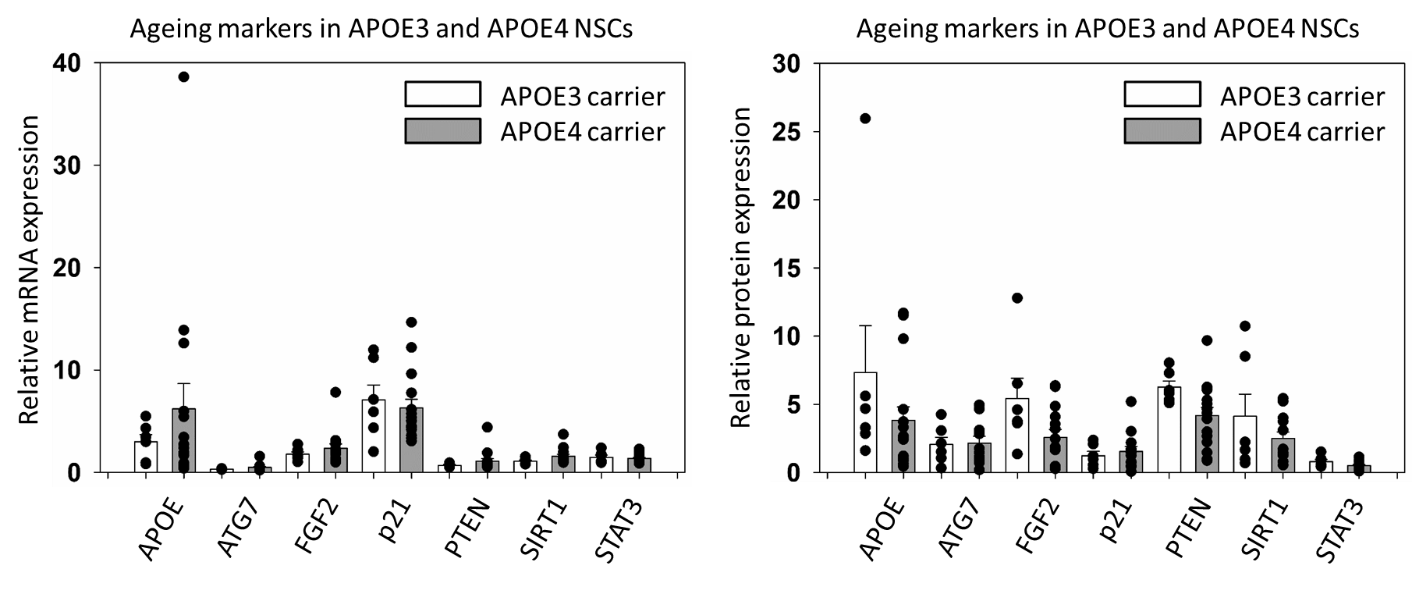
**

**Fig. S6** Bar charts including data points similar to bar charts shown in Fig. 5A and 5B.


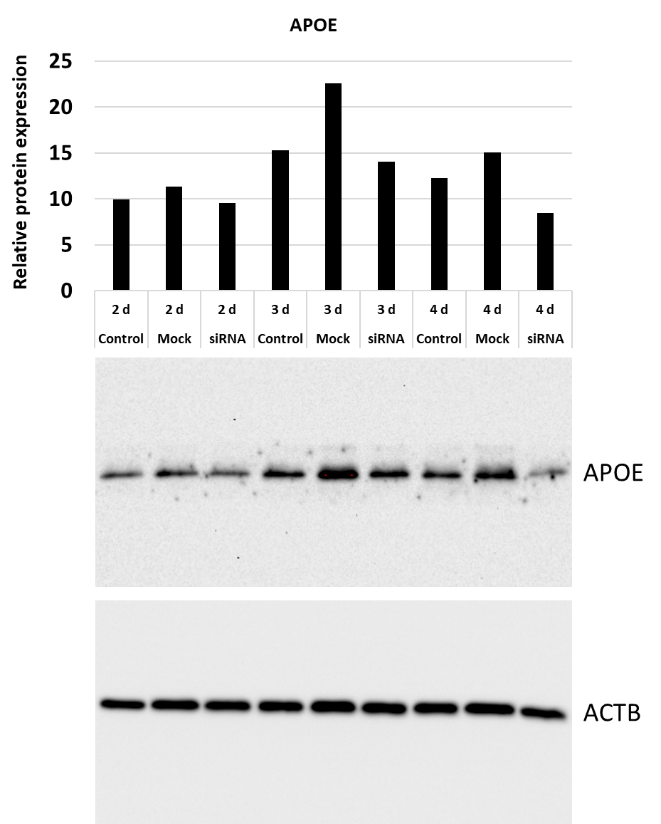

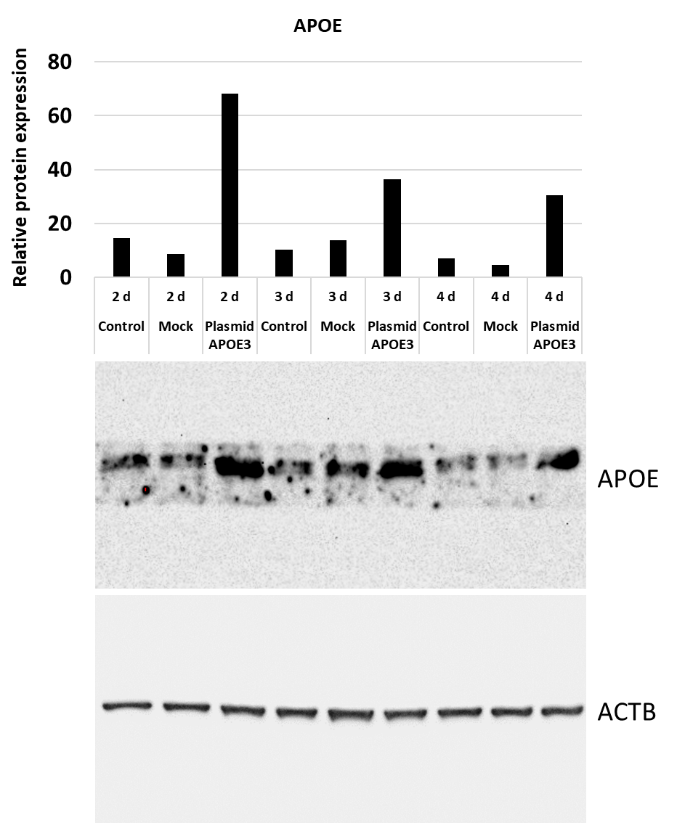


**Fig. S7** Apolipoprotein E (APOE) protein analysis in neural stem cells (NSCs) derived from healthy (reference) iPSCs WISCi004-B. NSCs were transfected with Apolipoprotein E (APOE) siRNAs and APOE3 plasmids and analyzed on 2 d, 3 d, and 4 d. Bar charts show WB analysis for APOE normalized to Actin beta (ACTB). Transfections are shown in comparison to mock transfections and control NSCs that were not treated. The related stained western blot (WB) membranes are shown below.


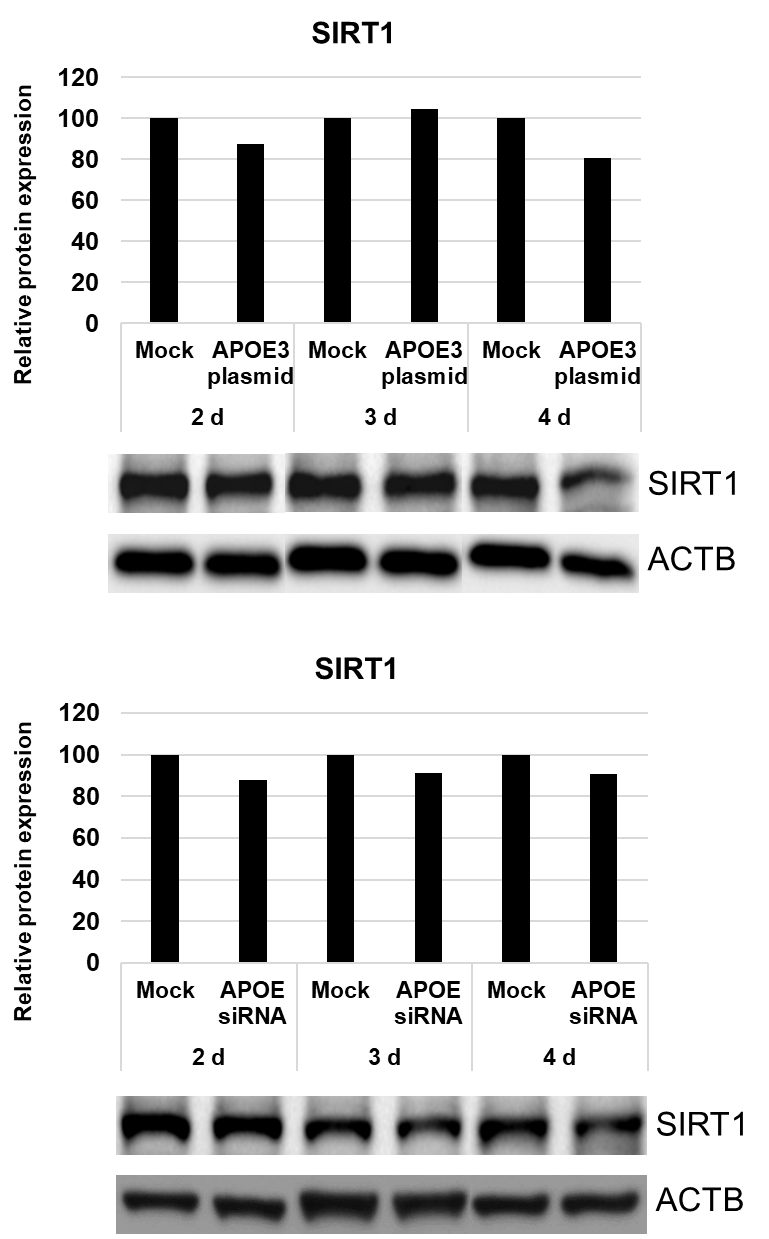


**Fig. S8** Sirtuin1 (SIRT1) protein analysis in neural stem cells (NSCs) derived from healthy (reference) iPSCs WISCi004-B. NSCs were transfected with Apolipoprotein E (APOE) siRNAs and APOE3 plasmids and analyzed on 2 d, 3 d, and 4 d. Bar charts show WB analysis for SIRT1 normalized to Actin beta (ACTB). Transfections are shown in comparison to mock transfections (set to 100). The related stained western blot (WB) membranes are shown below. Representative data of two independent experiments.
